# Reliability Analysis of Lithium-Ion Battery Aging Lifetime Based on Adaptive Progressive Type-II Censored Data

**DOI:** 10.3390/e28101108

**Published:** 2026-10-11

**Authors:** Shixiao Xiao, Jingwei Xu, Yanqiu Zeng, Xinyu Wu

**Affiliations:** Department of Mathematics and Digital Science, Chengyi College, Jimei University, Xiamen 361021, China

**Keywords:** power Rayleigh distribution, adaptive progressive Type-II censoring, maximum likelihood estimation, Bayesian inference, reliability quantification, lithium-ion battery

## Abstract

Lithium-ion battery accelerated aging tests are costly and use small samples, while conventional censoring ignores electrochemical degradation dynamics and biases lifetime estimates. The power Rayleigh distribution has a monotonically increasing hazard rate consistent with aging from solid electrolyte interphase thickening and lithium plating. We combine it with adaptive progressive Type-II censoring that stops early sample withdrawals once failure capacity crosses the end-of-life threshold, preserving right tail degradation information. Parameters are estimated by maximum likelihood with multiple starts and limited memory BFGS, and by hybrid Metropolis–Hastings Markov chain Monte Carlo Bayesian inference, validated by Monte Carlo simulation and NASA 4 °C cycling data. Mild censoring retains roughly 10% more right tail information than heavy censoring. For point estimation, maximum likelihood has slightly lower bias for the moderate sample (*n* = 66), whereas Bayesian estimation is more robust under small sample censoring (*n* = 40); the highest posterior density credible interval is on average 12% narrower than the asymptotic interval while keeping nominal coverage. Although this distribution reparametrizes the two-parameter Weibull family with identical fit indices, its Shannon entropy and quantile functions have elementary closed forms, avoiding nested numerical integration and delivering about 740 times faster Bayesian sampling per chain with better numerical stability for onboard computation. Sensitivity analysis shows removing one extreme cell weakens the fit, so the framework suits homogeneous cell subpopulations under uniform conditions. Validation remains limited to low-temperature cycling; extending it to combined temperature and charge rate stresses is our immediate priority.

## 1. Introduction

In the field of Prognostics and Health Management (PHM) for energy equipment, the aging lifetime and reliability analysis of lithium-ion batteries has been widely adopted as the core basis for energy storage safety assessment. Yang et al. [1] demonstrated through electrochemical modeling that long-cycle charge–discharge aging of lithium-ion batteries follows a two-stage degradation pattern: capacity decays linearly and steadily in the early stage, while side reactions such as lithium plating lead to an abrupt cliff-like capacity fade in the late stage. From a statistical lifetime perspective, battery lifetime follows a strongly right-skewed distribution, and failure risk increases monotonically with cycle number.

Current engineering models mostly adopt traditional lifetime distributions such as exponential, two-parameter Weibull, Gamma and lognormal distributions. However, Taketomi et al. [2] pointed out that these distributions have limited compatibility with the aging mechanism of lithium-ion batteries and therefore fail to capture the full-life-cycle capacity degradation pattern. It is therefore necessary to develop a dedicated lifetime distribution with a monotonically increasing hazard rate and a compact mathematical form.

Beyond the choice of lifetime distribution, recent years have seen rapid progress in data-driven, intelligent condition monitoring of rotating machinery and energy equipment. For example, multi-signal blind deconvolution has been proposed for fault feature enhancement of rolling element bearings under strong background noise [3], and a variational gated hierarchical memory network has been developed for rolling-bearing fault diagnosis in high-noise environments [4]. These signal-driven methods excel at extracting weak fault impulses from raw vibration data and have pushed diagnostic accuracy to a high level. Nevertheless, their outputs are typically point predictions of state or remaining useful life, and the accelerated aging tests used to generate such data remain limited by sample size and censoring cost. A complementary, statistics-based layer is therefore still required: given a censored accelerated-test sample, one must infer the lifetime distribution, its uncertainty interval and the reliability at a given capacity threshold. The present work targets this complementary layer rather than replacing the intelligent signal-processing layer; in a practical PHM pipeline the two are naturally combined as signal feature extraction followed by censored lifetime inference and uncertainty-aware reliability output.

In 2020, Bhat et al. [5] first proposed the power Rayleigh distribution (PRD) and derived its full mathematical properties. This two-parameter model can generate a monotonically increasing hazard rate curve solely through its shape parameter. Its probability density function, reliability function, hazard rate function and quantiles all have closed-form elementary expressions, which naturally conform to the aging characteristics of lithium-ion batteries: cumulative degradation over long cycles and a sharp rise in failure risk at the end of life. In the same year, Kilany et al. [6] verified the general fitting superiority of PRD for right-skewed lifetime data through empirical fitting on hydrological datasets. In 2022, Migdadi et al. [7] first extended the PRD to the ordinary Progressive Type-II Censoring (PC-II) scenario and completed full parameter inference simulations. Subsequently, Migdadi et al. [8] further improved upon the inherent limitations of PC-II, adapted the PRD to the Adaptive Progressive Type-II Censoring (APT-II) framework and systematically verified the modeling stability of the distribution under complex censored samples. The PRD is in fact a reparametrized special case of the two-parameter Weibull family; it cannot therefore out-fit Weibull, and its value lies in exposing elementary closed forms for the wear-out shape, the Shannon entropy and the quantile function, which is precisely what the battery Bayesian-inference and online-PHM modules below exploit.

Full-life-cycle battery cycling tests are time-consuming and incur high costs of cell materials and testing facilities. To shorten test cycles and reduce sample consumption, censored test schemes are widely adopted in engineering for accelerated aging lifetime tests of lithium-ion batteries. Among them, PC-II is a classic and widely used lifetime test scheme, which allows dynamic removal of surviving samples after each observed failure to balance test cost and sample information content. However, PC-II adopts fixed removal rules and cannot flexibly control the total test duration, which makes it difficult to meet the engineering requirement of preset shutdown thresholds in battery lifetime tests. To address this issue, Ng et al. [9] proposed the APT-II scheme, which incorporates a time-triggered shutdown constraint on the basis of failure-censored testing, making it more suitable for lithium-ion battery aging tests with limited resources and time budgets. Concretely, three engineering pain points of battery accelerated aging tests motivate APT-II rather than a fixed PC-II rule: (i) a fixed removal vector cannot adapt to the observed degradation pace, so a test designed for a fast-failing batch continues to occupy costly channel time long after surviving cells have already crossed the end-of-life (EOL) capacity threshold; (ii) ordinary Type-II censoring pre-fixes the number of observed failures but leaves test duration open-ended, which is incompatible with the fixed chamber booking and release schedules of cell qualification; and (iii) battery failure is naturally defined by a capacity/SOH threshold (here 0.8 Ah), so withdrawal decisions should be coupled to that threshold rather than to an arbitrary count. APT-II resolves these by freezing the early-removal rule once the observed failure capacity crosses the threshold and deferring the remaining withdrawals to the last observed failure, thereby bounding both test duration and sample cost while retaining right-tail degradation information.

Although the studies reviewed above have made important advances in PRD censored statistical inference, the existing literature exhibits several notable gaps. Migdadi et al. [8] conducted APT-II-based PRD modeling on general simulated datasets but did not address the targeted adaptation to measured lithium-ion capacity data and electrochemical aging mechanisms. Although Mustafa et al. [10] constructed a PRD-based accelerated lifetime model in 2026, they did not derive a coupled formulation that integrates the APT-II censoring scheme with the capacity degradation process of lithium-ion batteries. Consequently, the resulting model is not directly applicable to lifetime evaluation of energy-storage batteries under small-sample and short-cycle conditions.

To fill this gap, the present paper develops a comprehensive reliability analysis framework for energy storage cell aging lifetime based on APT-II lithium-ion battery aging data. The core research work is divided into two modules: We emphasize that the PRD, APT-II sampling, maximum likelihood estimation and Bayesian MCMC inference are established statistical tools; the novelty of this paper is not a new distribution or a new censoring theorem but their physics-coupled adaptation to measured lithium-ion battery aging data—coupling the PRD monotone hazard to the SEI-thickening/lithium-plating mechanism, deriving the APT-II joint likelihood for this battery setting, and validating the pipeline on NASA-measured data under the small-sample, threshold-censored conditions that battery qualification actually imposes.

1. Theoretical derivation and algorithm construction: The joint likelihood function of the PRD under APT-II censoring is derived, and two comparative censoring schemes for battery accelerated aging tests are designed. A multi-start L-BFGS-B constrained optimization algorithm is introduced to solve the maximum likelihood estimation (MLE) of model parameters, and a hybrid M-H Markov chain Monte Carlo (M-H MCMC) sampling framework is established. Bayesian estimation of battery lifetime is performed under three loss functions: squared error, generalized entropy and LINEX. Meanwhile, 95% asymptotic confidence intervals (ACIs), bootstrap confidence intervals (BCIs) and highest posterior density credible intervals (HPDs) are constructed to quantify the uncertainty of lifetime estimation.
2. Simulation validation and empirical demonstration: A comprehensive evaluation index system for lithium-ion battery aging lifetime is established. Based on Monte Carlo simulations with multiple sample sizes and multiple operating conditions, the accuracy of various lifetime estimation methods is compared horizontally in terms of estimation bias, root mean square error, interval coverage and average interval length. The K-S test, Akaike Information Criterion (AIC) and Bayesian Information Criterion (BIC) criteria are employed to conduct goodness-of-fit tests for the PRD model. Empirical analysis is carried out on a public dataset of lithium-ion battery cycle aging to evaluate the estimation performance of the proposed framework on real data and to compute battery reliability and remaining useful life under given capacity fade thresholds.

Based on the above two modules, the remainder of this paper is organized as follows. Section 2 systematizes the microscopic mechanism of lithium-ion battery capacity degradation and its compatibility with the PRD; derives the closed-form expressions of PRD probability density, reliability, hazard rate, and Shannon entropy; and elaborates the APT-II test mechanism and model evaluation index system. Section 3 constructs the complete PRD statistical inference framework: From the frequentist perspective, the joint log-likelihood function under APT-II censoring is derived, the parameter MLE is solved by the multi-start L-BFGS-B algorithm, the ACI is constructed based on the Delta method and the observed Fisher information matrix, and the BCI is generated by the bootstrap method. From the Bayesian perspective, a non-informative joint prior is selected, and hybrid M-H MCMC sampling is performed to obtain Bayesian point estimates under three loss functions and to construct the HPD interval with optimal compactness, realizing full-process uncertainty quantification from point estimation to interval estimation. Section 4 and Section 5 conduct cross-validation based on the NASA low-temperature lithium-ion battery dataset and Monte Carlo simulations, respectively, and compare the accuracy, stability and engineering practical value of various estimation methods under different sample sizes for the CS-I and CS-II censoring schemes. Section 6 summarizes the core conclusions and research limitations, and it prospects expansion directions such as multi-stress coupled accelerated lifetime modeling, competing risk analysis and lightweight Bayesian algorithms, providing theoretical and empirical support for research related to battery censored lifetime.

## 2. Theoretical Foundations and Preliminaries

### 2.1. Capacity Degradation Mechanism of Lithium-Ion Batteries and PRD Adaptability

**Figure 1:**
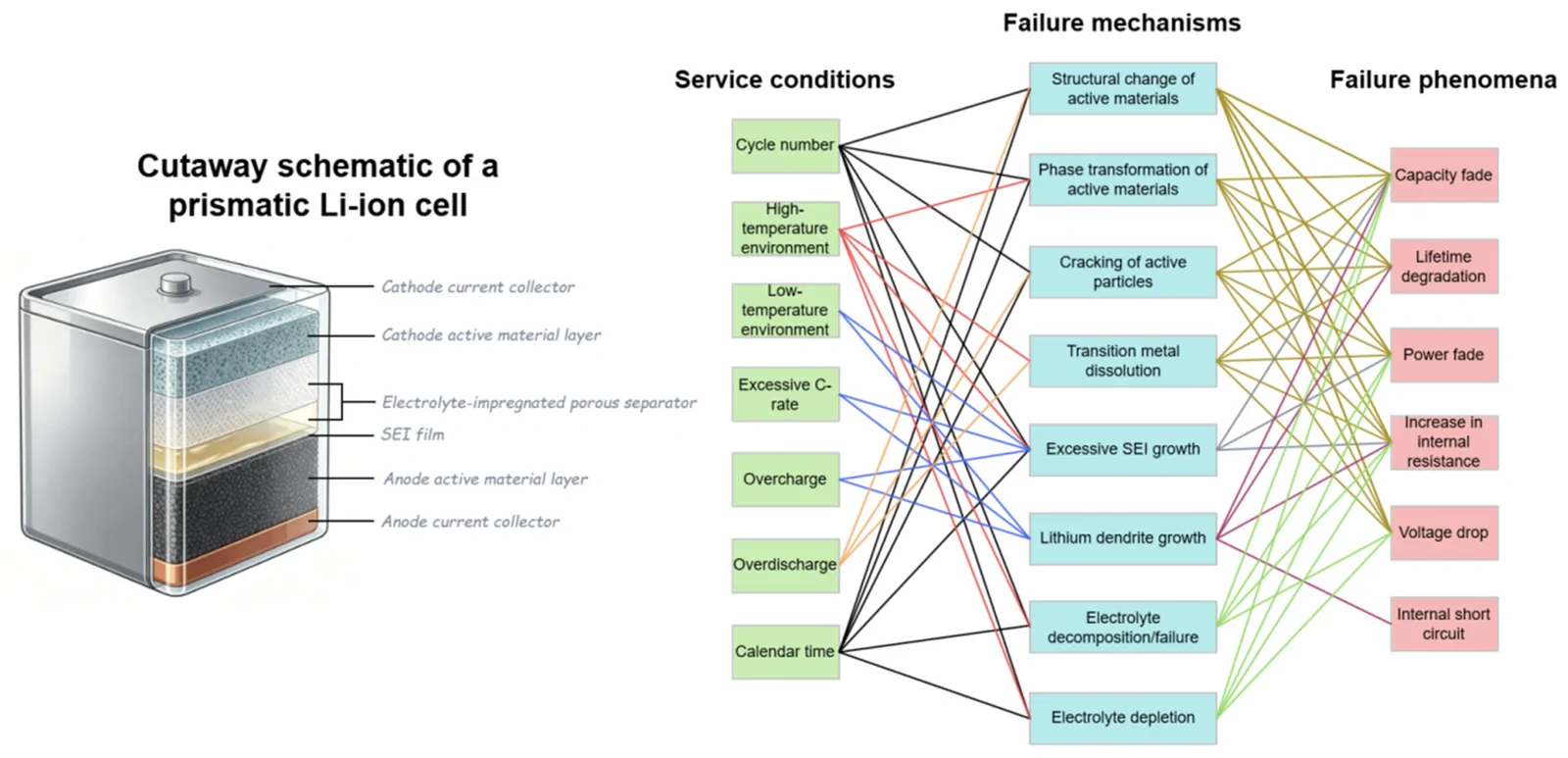
Schematic of multi-factor coupled failure mechanisms for lithium-ion batteries.

Under cyclic charge–discharge and long-term shelving conditions, multiple usage stresses and microscopic material degradation mechanisms of cells exhibit many-to-many coupling relationships [11], which continuously induce irreversible capacity fade. The coupling relationships among various factors are shown in Figure 1. This paper selects the discharge capacity per cycle as the quantitative indicator of cell lifetime, ignores secondary failure paths such as slight electrode expansion and local degradation of conductive agents, and focuses on the capacity fade process for reliability modeling.

Four core microscopic mechanisms causing cell capacity loss are summarized as follows:

- Repeated rupture and reformation of the solid electrolyte interphase (SEI) at the anode continuously consume cyclable lithium in the system; the thickening of the SEI film increases the electrode interface impedance, resulting in simultaneous attenuation of capacity and power. The evolution law of this interfacial side reaction has been verified by cycle tests [12].
- Repeated intercalation and deintercalation of lithium ions damages the crystal lattice of the cathode, inducing pulverization of active particles [13] and dissolution of transition metals [14]. The dissolved metals migrate to the anode surface, which not only reduces the effective active material of the cathode, but also easily pierces the separator and causes internal short circuits. The shift of the incremental capacity curve can quantitatively characterize the degree of such crystal degradation.
- High temperature and moisture accelerate the oxidative decomposition of the electrolyte [15]. Insufficient electrolyte reserve and lack of suitable additives continuously aggravate degradation. Electrolyte dry-out blocks lithium-ion transport channels, causes monotonically increasing internal resistance, and significantly reduces the long-cycle stability of cells; meanwhile, electrolyte decomposition generates a large amount of gas and accelerates cell failure [16].
- Low temperature, high-rate fast charging, excessively low N/P ratio and overcharging all induce lithium plating on the anode and the growth of lithium dendrites [17]. Continuous dendrite growth pierces the separator and forms local internal short circuits, accompanied by gas generation and local high temperature. Under extreme conditions, separator shrinkage induces thermal runaway, which brings major safety risks [18].

Under the superposition of multiple aging mechanisms, battery capacity presents a right-skewed distribution characteristic: slow decline in the early stage, accelerated decline in the middle stage, and a sharp drop in the late stage. This evolutionary behavior aligns with the strongly right-skewed lifetime distribution and sharply increasing late-stage hazard rate of the PRD, providing physical mechanism support for PRD-based battery lifetime modeling.

Let the random lifetime variable *X* follow the two-parameter PRD, denoted as *X*∼PRD(*θ*, *β*), where the scale parameter *θ* > 0 and the shape parameter *β* > 0. Its probability density function, cumulative distribution function, reliability function and hazard rate function are given by

(1) $$f\left(x;\theta ,\beta \right)=\frac{\beta }{\theta^{2}}x^{2\beta -1}\exp\left(-\frac{x^{2\beta }}{2\theta^{2}}\right),x>0$$

(2) $$F\left(x;\theta ,\beta \right)=1-\exp\left(-\frac{x^{2\beta }}{2\theta^{2}}\right),x>0$$

(3) $$R\left(x;\theta ,\beta \right)=1-F\left(x;\theta ,\beta \right)=\exp\left(-\frac{x^{2\beta }}{2\theta^{2}}\right),x>0$$

(4) $$h\left(x;\theta ,\beta \right)=\frac{f\left(x;\theta ,\beta \right)}{R\left(x;\theta ,\beta \right)}=\frac{\beta }{\theta^{2}}x^{2\beta -1},x>0$$

where the scale parameter *θ* regulates the overall baseline lifetime level of the battery, and the shape parameter *β* determines the evolution trend of the hazard rate. Taking the first derivative of the hazard rate function with respect to the lifetime variable yields $\frac{\partial h\left(x;\theta ,\beta \right)}{\partial x}=\frac{\beta \left(2\beta -1\right)}{\theta^{2}}x^{2\beta -2}$. Based on this derivative, we classify three types of hazard rate evolution patterns, which correspond to three typical aging conditions of lithium-ion batteries:

- When *β* < 0.5, ∂*h*/∂*x* < 0, and the hazard rate decreases monotonically with lifetime. This feature corresponds to the early failure scenario dominated by factory defects: defective cells with micro-cracks in electrodes or separator flaws fail intensively in the initial cycles, and the overall failure risk decreases continuously as defective samples are gradually eliminated.
- When *β* = 0.5, ∂*h*/∂*x* = 0, and the hazard rate is constant. This adapts to the ideal steady-state operation scenario without material loss or inherent manufacturing defects.
- When *β* > 0.5, ∂*h*/∂*x* > 0, and the hazard rate increases monotonically with lifetime, which is highly consistent with the aging characteristics of lithium-ion batteries under long-term cyclic cumulative damage. As the number of cycles increases, side reactions such as lithium plating, electrolyte decomposition and active material loss continue to intensify, and the failure risk rises rapidly when the battery enters the end of life.

**Figure 2:**
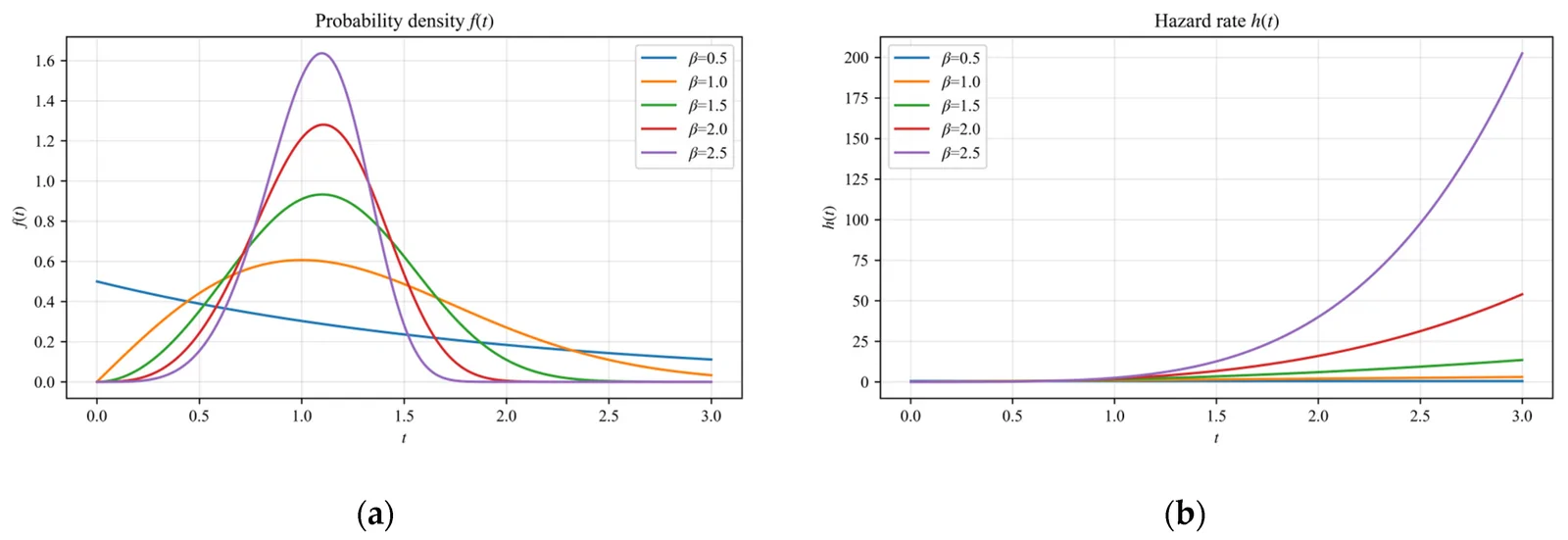
Probability density and hazard rate curves of PRD under different shape parameters. (**a**) probability density curves of the PRD under different shape parameters; (**b**) hazard rate curves of the PRD under different shape parameters.

With the scale parameter fixed at *θ* = 1.0, probability-density and hazard-rate curves for the PRD are plotted for *β* = {0.5, 1.0, 1.5, 2.0, 2.5}, as shown in Figure 2.

The left panel shows that as *β* increases, the peak of the probability density shifts to the right and the dispersion of the distribution increases. The distribution shape transitions from highly right-skewed to approximately symmetric, and the skewness can be flexibly adjusted by a single shape parameter without multi-parameter coordination. The right panel shows that all hazard rates corresponding to *β* > 0.5 increase monotonically with lifetime, and the larger *β* is, the faster the hazard rate rises.

It should be noted that the PRD is a special case of the two-parameter Weibull distribution: when the Weibull shape parameter *k* = 2*β* and the scale parameter $\lambda ={\left(2\theta^{2}\right)}^{\frac{1}{2\beta }}$, the PDF, CDF and hazard rate functions of the two are completely equivalent. However, for the lithium-ion battery APT-II scenario, the PRD form provides a more intuitive physical interpretation of the aging-related parameter *β*. Furthermore, as demonstrated in this paper, the PRD Shannon entropy admits a complete elementary closed form that avoids infinite series iteration, and its numerical stability is superior to the general Weibull entropy expression, which confers computational advantages in battery censored Bayesian iteration scenarios. Specifically, the generalized entropy loss function in Section 3 directly uses this closed-form construction, avoiding the defect that transforming Weibull distribution entropy requires infinite series solution and has convergence interval constraints. It still maintains global convergence under the strongly right-skewed limit of *β* → 0.5, which verifies the engineering application advantage of the PRD over Weibull from the perspective of calculation reliability. Concretely, the Weibull shape and scale parameters are obtained from the PRD parameters (*θ*, *β*) as *k* = 2*β* and $\lambda ={\left(2\theta^{2}\right)}^{\frac{1}{2\beta }}$. For the NASA estimate (*θ* = 0.77, *β* = 1.5989), this gives *k* = 3.198 and *λ* = 1.055, which exactly reproduce the fitted Weibull row in Table 1.

### 2.2. Higher-Order Statistical Properties of PRD

For any continuous lifetime random variable, Shannon entropy is defined as

(5) $$H=-\int_{0}^{\infty }f\left(x\right)\ln f\left(x\right)\mathrm{dx}$$

**Theorem 1.**  *Let the random variable X* ~ *PRD*(*θ*, *β*) *with parameter vector ϕ* = (*θ*, *β*)*. Then the Shannon entropy of PRD has the analytical expression*

(6) $$H\left(\phi \right)=-\int_{0}^{\infty }f\left(x;\theta ,\beta \right)\ln f\left(x;\theta ,\beta \right)\mathrm{dx}=-\ln\frac{\beta }{\theta^{2}}-\left(2\beta -1\right)E\left(\ln X\right)+E\left(\frac{X^{2\beta }}{2\theta^{2}}\right).$$

which contains two key expectation terms. First, for *E*(*X*^2*β*^), let *Y* = *X^β^* ~ Ray(*θ*); then, *X*^2*β*^ = *Y*^2^. The second moment of the Rayleigh distribution satisfies *E*(*Y*^2^) = 2*θ*^2^, so *E*(*X*^2*β*^) = 2*θ*^2^. Second, for *E*(ln*X*), from $X=Y^{\frac{1}{\beta }}$, taking logarithms on both sides gives $\ln X=\frac{1}{\beta }\ln Y$. The logarithmic expectation of a Rayleigh variable satisfies

(7) $$E\left(\ln Y\right)=\ln\theta +\frac{\ln2+\psi \left(\frac{1}{2}\right)}{2}=\ln\left(\sqrt{2}\theta \right)+\frac{\psi \left(\frac{1}{2}\right)}{2}$$

where *ψ*(•) is the digamma function, satisfying *ψ*(1) = −*γ* with *γ* ≈ 0.5772 being the Euler–Mascheroni constant. Substituting the transformation relation yields

(8) $$E\left(\ln X\right)=\frac{\ln\left(2\theta^{2}\right)}{2\beta }+\frac{\psi \left(\frac{1}{2}\right)}{2\beta }$$

The derivation strategy for the Shannon entropy of a two-parameter lifetime distribution, which expresses the entropy as a function of *E*(ln*T*) and *E*(*T*^−*τ*^), has been successfully applied to other prominent lifetime models as well. For instance, Ren and Hu [19] employed an analogous approach to derive the Shannon and Rényi entropies of the inverse Weibull distribution, another widely used model in reliability analysis. This methodological consistency across distinct distributional families validates the analytical steps undertaken here and underscores the general applicability of the entropy derivation framework to right-skewed lifetime models.

Substituting the results of *E*(*X*^2*β*^) and *E*(ln*X*) back into Equation (6) and combining like logarithmic terms, the complete closed form of PRD Shannon entropy is obtained as

(9) $$H\left(\phi \right)=-\ln\frac{\beta }{\theta^{2}}-\frac{2\beta -1}{2\beta }\ln\left(2\theta^{2}\right)-\frac{2\beta -1}{2\beta }\psi \left(\frac{1}{2}\right)+1$$

**Remark 1.**  *This entropy closed form converges globally for any β* > 0*. When β* = 0.5*, the cross logarithmic term disappears and the entropy expression reduces to a constant offset form. When β* = 1*, PRD degenerates to the standard Rayleigh distribution, and substitution gives the Shannon entropy of the Rayleigh distribution. Compared with the transformed Weibull distribution entropy (which requires infinite series solution and has convergence interval constraints), the PRD entropy can be computed directly via its analytical closed form, with superior numerical stability. The Bayesian generalized entropy loss function in the following section will be directly constructed using this closed form to reduce the numerical solution complexity in the parameter estimation process.*

It is noteworthy that Bayesian inference for Shannon entropy under progressive censoring has recently attracted considerable attention in the reliability literature. For instance, Zeng et al. [20] developed a comprehensive Bayesian framework for estimating the Shannon entropy of the transmuted Weibull distribution under Progressive Type-II censored samples, utilizing a hybrid Gibbs sampling algorithm within the MCMC framework. Their results demonstrated that Bayesian estimators consistently outperform their maximum likelihood counterparts in terms of bias and mean squared error, particularly under small-sample and heavy-censoring scenarios. In contrast to their setting, where the entropy expression involves an infinite series expansion that necessitates numerical integration within each MCMC iteration, the closed-form entropy of the PRD derived in Theorem 1 completely avoids such approximation. This analytical tractability not only enhances numerical stability, particularly under the strongly right-skewed limit of *β* → 0^+^, but also substantially reduces the computational overhead in the Bayesian iterative procedures employed in Section 3.3, thereby offering a practical advantage for online battery health prognosis applications.

For *X*∼PRD(*θ*, *β*), let *Y* = *X^β^* ~ Ray(*θ*); then, the closed form of the r-th raw moment can be obtained as

(10) $$E\left[X^{r}\right]=E\left[Y^{\frac{r}{\beta }}\right]={\left(2\theta^{2}\right)}^{\frac{r}{2\beta }}\Gamma \left(\frac{2\beta +r}{2\beta }\right)$$

setting *r* = 1 gives the lifetime mean

(11) $$E\left[X\right]={\left(2\theta^{2}\right)}^{\frac{1}{2\beta }}\Gamma \left(\frac{2\beta +1}{2\beta }\right)$$

the variance is calculated by the definition Var(*X*) = *E*[*X*^2^] − (*E*[*X*])^2^, substituting *r* = 2 to solve the second raw moment. The variance has no simplified elementary closed form; high-precision numerical integration is used in engineering calculation, and the standard deviation is the arithmetic square root of the variance.

Let the cumulative distribution satisfy *F*(*x_p_*) = *p*. Solving Equation (2) inversely gives the *p*-th quantile

(12) $$x_{p}={\left[-2\theta^{2}\ln\left(1-p\right)\right]}^{\frac{1}{2\beta }},0<p<1$$

the quantile can be used to quantitatively calculate the remaining cycle life of the battery under a specified reliability, as well as the failure cycle number corresponding to the capacity fade threshold. This formula will be used in Section 4 to calculate the failure capacity quantile corresponding to the EOL = 1.0 Ah threshold, so as to realize quantitative evaluation of remaining useful life.

### 2.3. Adaptive Progressive Type-II Censoring Scheme

In a lifetime test, *n* independent and identically distributed samples are placed on test, and the number of failure samples to be observed *m* is preset. When the *i*-th failure is observed, *R_i_* surviving units are randomly withdrawn from the remaining unfailed samples. Finally, ordered failure observations *x*_1:*m*:*n*_ < *x*_2:*m*:*n*_< ⋯ <*x_m_*_:*m*:*n*_ are obtained. This data collection scheme is the standard ordinary Progressive Type-II censoring, which only specifies the number of observed failures without an upper limit on test time. The systematic theoretical foundation and review of progressive censoring methodology can be found in the monograph by Balakrishnan [21].

**Figure 3:**
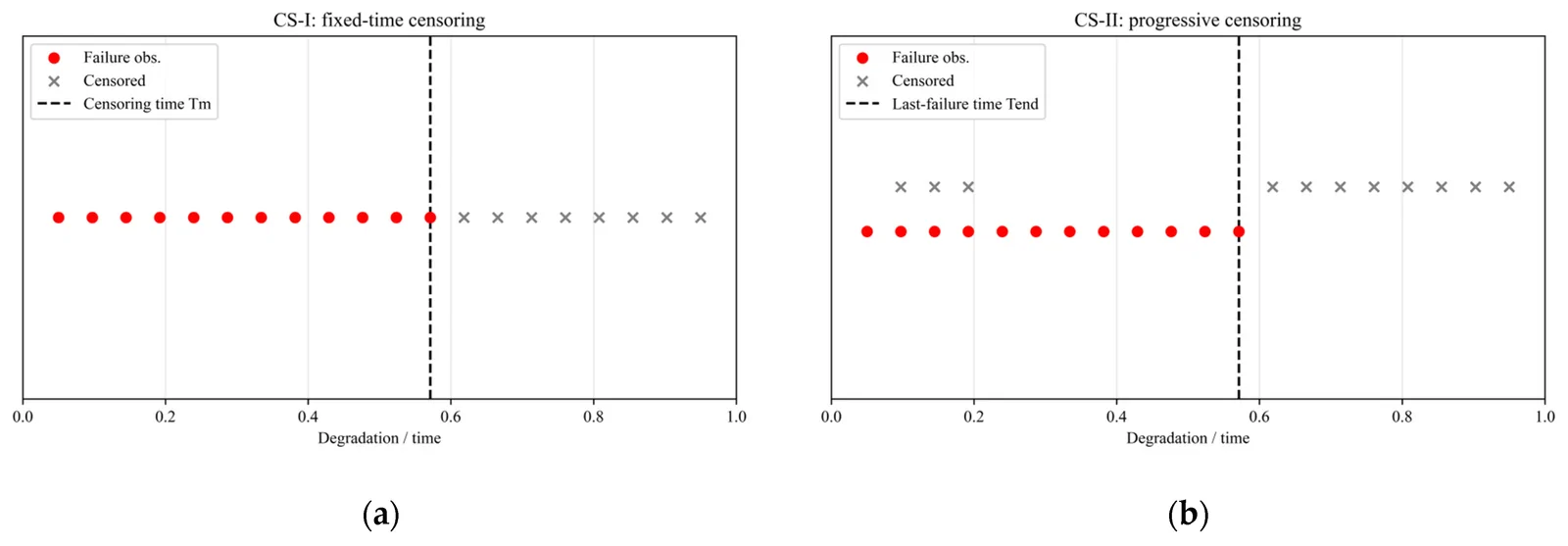
Schematic diagram of CS-I and CS-II removal sequences. (**a**) removal sequence under the CS-I scheme; (**b**) removal sequence under the CS-II scheme.

This paper adopts the APT-II framework proposed by Ng et al. [9], adding a capacity shutdown threshold *T* constraint on the basis of conventional progressive removal. Define the critical failure index *J* = max{*j*: *x_j_*_:*m*:*n*_ < *T*}, which represents the last set of failure observations with capacity below the shutdown threshold. The piecewise adaptive removal rule is as follows: when *j* ≤ *J*, execute the preset removal number *R_i_*; when *J* < *j* < *m*, stop early removal and set *R_i_* = 0; when *j* = *m*, the final removal amount satisfies $R_{m}=n-m-\sum_{j=1}^{J}R_{i}$. This scheme simultaneously constrains the number of failures and the capacity boundary, balancing test cost and sample information. The censoring timing rule is shown in Figure 3.

The unified basic test parameters are as follows: total sample size *n* = 66, preset number of failure observations *m* = 40, capacity judgment threshold *T* = 0.8 Ah, total samples to be removed in advance *n* − *m* = 26, and critical failure index *J* = 26, satisfying *J* < 40, so the adaptive removal rule can take effect normally.

Two sets of differentiated removal sequence comparative tests are designed. The CS-I sequence is $\left(2,\overbrace{0\times 20},2,\overbrace{0\times 17},22\right)$: 2 samples are removed after the 1st and 22nd failures, respectively, no removal is performed after the 2nd to 21st and 23rd to 39th failures, and 22 samples are removed all at once after the last failure. This scheme features small early removal and batch removal at the end, suitable for scenarios with high requirements on sample information and loose test duration constraints. The CS-II sequence is $\left(8,\overbrace{0\times 25},8,\overbrace{0\times 10}\right)$: 8 samples are removed after the 1st and 27th failures, respectively, no removal is performed after the 2nd to 26th and 28th to 39th failures, and 10 samples are removed all at once after the last failure. This scheme removes a large number of samples in the early stage, greatly shortening the overall test cycle, and is suitable for scenarios with high test cost and strict duration constraints.

The two schemes only differ in the intensity of early removal as a single variable. Complete parameter inference and simulation comparison will be performed based on the two sets of censored samples in the following sections.

### 2.4. Model Evaluation Index System

The evaluation indicators in this paper are divided into three categories: distribution goodness-of-fit indicators, parameter estimation accuracy indicators and interval evaluation indicators, which are uniformly defined here.

The distribution goodness-of-fit indicators include three types:

- Kolmogorov-Smirnov (K-S) test statistic $D=\sup\left|F_{n}\left(x\right)-\hat{F}\left(x\right)\right|$, where *F_n_*(*x*) is the empirical distribution function. The higher the test *p*-value, the better the PRD fitting effect; when *p* > 0.05, the fitting hypothesis cannot be rejected.
- Log-likelihood value $I\left(\hat{\theta },\hat{\beta }\right)$: the larger the value, the stronger the model’s ability to explain the sample.
- AIC and BIC criteria: AIC = −2𝓁 + 2*k*, BIC = −2𝓁 + *k* ⋅ ln(*n*), where *k* is the number of model parameters. Smaller values of both indicators indicate better generalization performance.

The parameter estimation accuracy indicators also include three types:

- Relative estimation efficiency $\mathrm{RE}=1-\frac{\left|{\hat{\theta }}_{\mathrm{censored}}-{\hat{\theta }}_{\mathrm{complete}}\right|}{\left|{\hat{\theta }}_{\mathrm{complete}}\right|}$, which quantifies the information retention degree of the censoring scheme. The closer to 1, the smaller the estimation bias and the lower the information loss.
- Bias: $\mathrm{Bias}\left(\hat{\phi }\right)=\frac{1}{N}\sum_{S=1}^{N}\left({\hat{\phi }}_{S}-\phi_{\mathrm{true}}\right)$, representing the systematic deviation of estimation. The closer to 0, the higher the accuracy.
- Root mean square error: $\mathrm{RMSE}\left(\hat{\phi }\right)=\sqrt{\frac{1}{N}{\sum_{S=1}^{N}\left({\hat{\phi }}_{S}-\phi_{\mathrm{true}}\right)}^{2}}$, where *N* is the number of simulation replications. It comprehensively measures bias and random fluctuation; the closer to 0, the higher the accuracy.

The interval evaluation indicators include the following:

- Coverage probability (CP): the closer to 95%, the better the effect.
- Average interval length (ALI): the smaller the value, the lower the estimation uncertainty.

## 3. Statistical Inference Methods for PRD Parameters

### 3.1. Maximum Likelihood Estimation for Adaptive Censored Samples

#### 3.1.1. Construction of Joint Log-Likelihood Function

Based on the classical theoretical framework of industrial lifetime data analysis [22], this section systematically derives PRD parameters under adaptive censoring. More recently, Xiao et al. [23] systematically developed maximum likelihood and Bayesian estimation procedures for the lifetime performance index of the generalized inverse Lindley distribution under the identical adaptive progressive Type-II censoring scheme. Their work demonstrated the effectiveness of combining numerical optimization, specifically the Newton–Raphson algorithm, with MCMC-based Bayesian inference for complex censored lifetime data. They further validated the construction of asymptotic, bootstrap, and highest posterior density intervals for quantifying estimation uncertainty. This successful application of the same analytical pipeline to a distinct lifetime model reinforces the generality and robustness of the inferential strategy adopted in our study, which we now tailor to the PRD framework for lithium-ion battery reliability quantification. Suppose *n* cells of the same PRD specification are initially put into the lifetime test, and the target number of failure observations *m* and the capacity shutdown threshold *T* are preset. The ordered failure observation sequence is denoted as *x*_1_ < *x*_2_ < ⋯ < *x_m_*, the adaptive piecewise removal vector is $R^{*}=\left(R_{1}^{*},R_{2}^{*},\cdots ,R_{m}^{*}\right)$, and the critical failure index is *J* = max{*j*: *x_j_*_:*m*:*n*_ < *T*}.

Before the *i*-th failure occurs, *i* − 1 failures and removals have been completed, and the number of remaining intact cells on the test bench can be written as

(13) $$n_{i}=n-\sum_{k=1}^{i-1}\left(R_{k}+1\right)$$

The combinatorial counting constant of the progressive censoring test is obtained by multiplying the remaining sample size at each failure node, corresponding to all combination possibilities of sample selection under different removal schemes:

(14) $$C=\prod_{i=1}^{m}n_{i}$$

The APT-II joint likelihood is composed of the product of *m* failure unit probability densities and the reliability of surviving units removed in each round:

(15) $$L\left(\theta ,\beta \right)=C\cdot \prod_{i=1}^{m}f\left(x_{i}\right)\cdot {\prod_{i=1}^{m}\left[R\left(x_{i}\right)\right]}^{R_{i}}$$

Substituting Equations (1) and (3) into this formula and combining all exponential terms gives the complete joint likelihood expression. Taking the natural logarithm yields the core log-likelihood function:

(16) $$l\left(\theta ,\beta \right)=m\ln\beta -2m\ln\theta +\left(2\beta -1\right)\sum_{i=1}^{m}\ln x_{i}-\frac{1}{2\theta^{2}}\sum_{i=1}^{m}\left(1+R_{i}\right)x_{i}^{2\beta }$$

This formula serves both as the objective function for multi-start L-BFGS-B optimization and as the kernel for constructing the MCMC Bayesian posterior distribution, providing a unified basis for the two parameter inference methods described later.

#### 3.1.2. Parameter Optimization Based on Multi-Start L-BFGS-B

The log-likelihood function of PRD is nonlinearly coupled with respect to the scale parameter *θ* and the shape parameter *β*. Setting the gradient ∇*I*(*θ*, *β*) = 0 admits no closed-form analytical solution. Therefore, this paper uses constrained L-BFGS-B with parameter boundary constraints for numerical optimization, adapting to the feasible domain constraints of *θ* > 0 and *β* > 0.

Taking the first partial derivatives with respect to *θ* and *β* respectively gives the gradient components of the log-likelihood function:

(17) $$\frac{\partial l}{\partial \theta }=-\frac{2m}{\theta }+\frac{1}{\theta^{3}}\sum_{i=1}^{m}\left(1+R_{i}\right)x_{i}^{2\beta }$$

(18) $$\frac{\partial l}{\partial \beta }=\frac{m}{\beta }+2\sum_{i=1}^{m}\ln x_{i}-\frac{1}{\theta^{2}}\sum_{i=1}^{m}\left(1+R_{i}\right)x_{i}^{2\beta }\ln x_{i}$$

The log-likelihood surface may have multiple local extrema, and a single initial point is prone to converge to a local optimum. To address this issue, we introduce a multi-start parallel iteration scheme:

1. Twenty-five sets of initial parameter combinations are uniformly generated on a grid within the feasible parameter domain;
2. Perform L-BFGS-B iteration independently for each set of initial values, with the convergence threshold set to 10^−8^ and the maximum number of iteration steps set to 5000;
3. After all iterations are completed, select the set of parameters with the smallest negative log-likelihood as the global optimal MLE solution.

The Hessian matrix of the log-likelihood function is numerically approximated via the central difference method, and the observed Fisher information matrix is then obtained as its negative:

(19) $$I\left(\hat{\theta },\hat{\beta }\right)=-E{\left[\frac{\partial^{2}l}{\partial \phi_{i}\partial \phi_{j}}\right]}_{\phi =\hat{\phi }}$$

where the asymptotic covariance matrix of the MLE parameters is the inverse of the Fisher information matrix, and the square root of the diagonal elements of the matrix gives the respective parameter standard errors, providing support for the derivation of the Delta-method asymptotic confidence interval.

#### 3.1.3. Existence and Uniqueness of Maximum Likelihood Estimators

**Theorem 2.**  *Let the ordered failure observations x*_1_ < *x*_2_ < ⋯ < *x_m_ be an APT-II sample from PRD*(*θ*, *β*)*. For any fixed value of the shape parameter β, the log-likelihood function I*(*θ*, *β*) *has a unique maximum point with respect to θ, i.e., the maximum likelihood estimator of θ exists and is unique.*

**Theorem 3.**  *Under the same conditions as Theorem 2, for any fixed value of the scale parameter θ, the log-likelihood function I*(*θ*, *β*) *has a unique maximum point with respect to β, i.e., the maximum likelihood estimator of β exists and is unique.*

**Proof of Theorem 2.**  Fix the shape parameter *β*, and introduce the scale parameter transformation $\tau =\frac{1}{\theta^{2}}\left(\tau >0\right)$. Then, the original log-likelihood function can be rewritten in terms of *τ*:

$$l\left(\tau \right)=m\ln\beta +\left(2\beta -1\right)\sum_{i=1}^{m}\ln x_{i}+m\ln\tau -\frac{\tau }{2}\sum_{i=1}^{m}\left(1+R_{i}\right)x_{i}^{2\beta }$$

Taking the first and second partial derivatives with respect to *τ* gives

(20) $$\frac{\partial l}{\partial \tau }=\frac{m}{\tau }-\frac{1}{2}\sum_{i=1}^{m}\left(1+R_{i}\right)x_{i}^{2\beta }$$

(21) $$\frac{\partial^{2}l}{\partial \tau^{2}}=-\frac{m}{\tau^{2}}<0$$

The second partial derivative is always less than 0 in the domain *τ* > 0, indicating that 𝓁(*τ*) is strictly concave in *τ*, and the first partial derivative is strictly monotonically increasing with respect to *τ*. For the boundary limits: as *τ* → 0^+^, ∂𝓁/∂*τ* → +∞; as *τ* → +∞, ∂𝓁/∂*τ* → −∞. By the intermediate value theorem for continuous functions, the first partial derivative has a unique root, i.e., the MLE of *τ* exists and is unique, corresponding to the unique MLE of the original scale parameter *θ*. □

**Proof of Theorem 3.**  Fix the scale parameter *θ*. The first partial derivative of the log-likelihood function with respect to *β* has been given by Equation (18). Further taking the second partial derivative

(22) $$\frac{\partial^{2}l}{\partial \beta^{2}}=-\frac{m}{\beta^{2}}-\frac{2}{\theta^{2}}\sum_{i=1}^{m}\left(1+R_{i}\right)x_{i}^{2\beta }{\left(\ln x_{i}\right)}^{2}$$

both terms on the right-hand side are negative, so ∂^2^𝓁/∂*β*^2^ < 0 holds identically in the domain *β* > 0, indicating that 𝓁(*β*) is strictly concave in *β*, and the first partial derivative is strictly monotonically decreasing with respect to *β*. For the boundary limits: as *β* → 0^+^, *m*/*β* → +∞ while the remaining terms are bounded, so ∂𝓁/∂*β* → +∞; as *β* → +∞, $x_{i}^{2\beta }$ grows exponentially and the negative term dominates, so ∂𝓁/∂*β* → −∞. By the intermediate value theorem, the first partial derivative has a unique root, i.e., the MLE of the shape parameter *β* exists and is unique. □

In summary, the log-likelihood function is strictly concave along both parameter coordinate directions, and the single-parameter maximum likelihood estimator exists and is unique. It should be noted that coordinate-wise strict concavity is not equivalent to joint strict concavity over the full parameter space; therefore, this paper does not claim an analytical proof of the global unique maximum of the joint likelihood. However, in all subsequent Monte Carlo simulations and measured data calculations, 25 sets of multi-start L-BFGS-B iterations all converge to the same parameter solution, which numerically supports the conclusion of a globally unique maximum.

#### 3.1.4. Asymptotic Confidence Intervals Based on the Delta Method

Based on the MLE asymptotic normality theory, the unknown parameter vector $\hat{\phi }={\left(\hat{\theta },\hat{\beta }\right)}^{T}$ satisfies the asymptotic distribution

(23) $$\hat{\phi }\;\sim \;N\left(\phi_{\mathrm{true}},I{\left(\hat{\phi }\right)}^{-1}\right)$$

where $I{\left(\hat{\phi }\right)}^{-1}$ is the covariance matrix, and its diagonal elements are the parameter variances. Given the 95% confidence level, the standard normal quantile *z*_0.975_ ≈ 1.96, the ACI for parameter *ϕ_k_* is

(24) $${\hat{\phi }}_{k}\pm z_{0.975}\cdot \sqrt{\mathrm{diag}{\left(I{\left(\hat{\phi }\right)}^{-1}\right)}_{k}}$$

For the reliability function *R*(*t*), the Delta method is used to linearly propagate the parameter variance: let *g*(*ϕ*) = *R*(*t*) with gradient vector ∇*g*, then the asymptotic variance of reliability is

(25) $$\mathrm{Var}\left(\hat{R}\left(t\right)\right)=\nabla g{\left(\hat{\phi }\right)}^{T}\cdot I{\left(\hat{\phi }\right)}^{-1}\cdot \nabla g\left(\hat{\phi }\right)$$

Finally, the 95% ACI for reliability is

(26) $$\hat{R}\left(t\right)\pm z_{0.975}\cdot \sqrt{\mathrm{Var}\left(\hat{R}\left(t\right)\right)}$$

### 3.2. Parametric Bootstrap Confidence Intervals

Asymptotic normal intervals rely on the large-sample asymptotic assumption and are prone to interval deviation and negative lower bounds for small-sample censored data. The parametric bootstrap resampling method is introduced to construct BCI, which does not rely on the normality assumption and is suitable for lithium-ion battery scenarios with limited censored samples.

The bootstrap resampling procedure is as follows: take the MLE estimates $\left(\hat{\theta },\hat{\beta }\right)$ from the original adaptive censored sample as the true parameters, strictly replicate the removal sequence rules of CS-I and CS-II, and generate *B* = 1000 sets of APT-II pseudo-samples. For each set of pseudo-samples, the same multi-start L-BFGS-B algorithm is used to solve a set of bootstrap parameter estimates and corresponding reliability estimates. All bootstrap estimates are sorted in ascending order, and the 2.5% and 97.5% quantiles of the sorted sequence are taken as the upper and lower bounds of the 95% BCI.

BCI makes full use of the true characteristics of the sample distribution and avoids normal approximation bias, but resampling introduces additional random fluctuations, and the average interval length is usually larger than that of HPD. It is suitable as a comparative verification benchmark.

### 3.3. Bayesian Estimation Method

#### 3.3.1. Prior Distribution and Posterior Density Function

This paper selects a non-informative joint prior to avoid subjective bias. The scale parameter and the shape parameter are independently set with prior distributions: the scale parameter takes the non-informative prior *π*(*θ*)∝1/*θ*, and the shape parameter takes the flat prior *π*(*β*)∝1. The joint prior is

(27) $$\pi \left(\theta ,\beta \right)\propto \frac{1}{\theta },\theta >0,\beta >0$$

Combined with the joint likelihood function in Equation (16), the joint posterior density of the parameters is obtained by Bayes’ theorem

(28) $$\pi \left(\theta ,\beta |t\right)=\frac{L\left(\theta ,\beta \right)\cdot \pi \left(\theta ,\beta \right)}{\int_{0}^{\infty }\int_{0}^{\infty }L\left(\theta ,\beta \right)\cdot \pi \left(\theta ,\beta \right)d\theta d\beta }$$

This posterior density has no elementary closed-form analytical solution and cannot be directly integrated to obtain Bayesian estimates. Therefore, the hybrid M-H MCMC sampling algorithm is used to realize the numerical solution of the posterior distribution.

#### 3.3.2. Hybrid M-H MCMC Sampling Algorithm

The iteration parameters are set as follows: total iterations 12,000, with the burn-in period discarding the first 2000 iterations to eliminate initial value deviation, retaining 10,000 effective posterior samples. The standard deviation of the bivariate normal proposal distribution matches the coupling degree of PRD parameters, and the acceptance rate is controlled within the optimal interval of 0.23–0.44.

The single-step M-H update procedure: given the current iteration parameters (*θ*^(*t*)^, *β*^(*t*)^), generate candidate parameters (θ*, β*) from the bivariate normal proposal distribution *q*(•∣*θ*^(*t*)^, *β*^(*t*)^); calculate the acceptance probability

$$\alpha =\min\left\{1,\frac{\pi \left(\theta^{*},\beta^{*}|t\right)}{\pi \left(\theta^{\left(t\right)},\beta^{\left(t\right)}|t\right)}\right\}$$

and generate a uniform random number *u* ~ *U*(0, 1); if *u* ≤ *α*, update the parameters to the candidate values, otherwise keep the current parameters. Repeat the iteration until the preset total number of iterations is reached, and output the effective posterior sample chain after burn-in.

Convergence criteria include the following: no persistent drift or periodic trend in the parameter trace plot, and a stable unimodal shape of the posterior histogram; meanwhile, report the Effective Sample Size (ESS) and the Gelman–Rubin diagnostic statistic $\hat{R}$. The M-H sampling acceptance rate falls within the optimal interval (0.234, 0.44). In this paper, the acceptance rate of CS-I is 0.3180 and that of CS-II is 0.3028; the $\hat{R}$ values of both schemes are close to 1.00, indicating good mixing of the sampling chain. However, due to the strong skewness and strong autocorrelation of the target posterior under heavy censoring, the ESS of the scale parameter is about 357–459, and the ESS of the shape parameter drops to 94–191, which meets the basic convergence threshold of ESS > 90 for the shape parameter. The posterior mean estimation error is within an acceptable range, meeting the convergence requirement.

#### 3.3.3. Bayesian Point Estimation Under Multiple Loss Functions

Based on the MCMC effective posterior samples, Bayesian estimates of parameters and reliability are solved under symmetric and two types of asymmetric loss functions, respectively:

- Squared error loss: A symmetric loss function. The optimal estimator is the posterior mean ${\hat{\phi }}_{\mathrm{SE}}=E\left(\phi |\mathrm{data}\right)=\frac{1}{S}\sum_{s=1}^{S}\phi_{s}$ where *S* = 1000 is the total number of effective posterior samples.
- Generalized entropy loss: An asymmetric loss function that imposes stronger penalty for overestimation errors, suitable for safety-conservative assessment scenarios of energy storage batteries. Its optimal estimator is the reciprocal of the −1/*c*-th power of the posterior ${\hat{\phi }}_{\mathrm{GE}}={\left(\frac{1}{N}\sum_{i=1}^{N}e^{-c\phi_{i}}\right)}^{-1/c}$, where *c* = 0.5 is the shape parameter of the loss function.
- LINEX loss: An asymmetric loss function with low cost for small overestimation and high penalty for large underestimation. The optimal estimator is the logarithmic transformation of the posterior exponential expectation ${\hat{\phi }}_{\mathrm{LINEX}}=-\frac{1}{c}\ln\left[\frac{1}{N}\sum_{i=1}^{N}e^{-c\phi_{i}}\right]$, where *c* = 0.5 is the shape parameter of the loss function.

#### 3.3.4. Highest Posterior Density Credible Interval (HPD)

The 95% HPD is constructed based on the sorted MCMC posterior samples. The core rule is to traverse all continuous 95% quantile sample windows and select the upper and lower bounds corresponding to the window with the smallest interval length as the HPD. Compared with ACI and BCI, HPD makes full use of the complete information of the posterior distribution, and it has the best interval compactness while ensuring the coverage probability close to the nominal level. It can quantify the complete uncertainty of parameter estimation and is suitable as the preferred interval estimation method for lithium-ion battery reliability assessment.

## 4. Empirical Analysis Based on NASA Low-Temperature Cycle

### 4.1. Dataset Source, Test Conditions and Preprocessing

This study adopts the public low-temperature cycle aging dataset from NASA Prognostics Center of Excellence (PCoE). Four lithium-ion cells numbered B0049 to B0052 are selected, all tested at a constant temperature of 4 °C, with differentiated discharge cut-off voltages set to 2.7 V, 2.5 V, 2.2 V and 2.0 V, respectively. The discharge capacity per cycle is recorded, and the discharge capacity is taken as the pseudo-lifetime degradation indicator of the battery. Reliability is defined as the probability that the cell discharge capacity is higher than the performance threshold. All four cells share the same chemistry and are cycled at the same 4 °C thermal condition and charge/discharge C-rate; only the discharge cutoff voltage varies across cells as a controlled cell-level condition. The pooled 66 observations therefore constitute one homogeneous subpopulation, and we deliberately do not pool across temperatures or C-rates.

Multi-step data cleaning is performed on the raw experimental data: first, remove invalid cycle records with empty discharge capacity or capacity lower than 0.1 Ah; second, delete non-physical abnormal outliers with a single capacity rebound greater than 2.0 Ah; merge valid samples of the four cells, and finally obtain 66 sets of valid degradation observation data for subsequent modeling analysis. The statistical characteristics of each cell sample are shown in Table 2. Cell B0052 is retained in the primary analysis rather than removed a priori: it is one of the four identical-chemistry NASA cells cycled at the same 4 °C, and its only four valid records reflect its 2.0 V discharge cutoff reaching the EOL threshold quickly, not a corrupt measurement. Because such a short, far-right-tail cell can dominate the estimated shape, we additionally perform a leave-one-out influence analysis in Section 4.7. This also suggests a pre-processing rule for the reliability pipeline: cells are first grouped by chemistry and operating condition (temperature, C-rate and cutoff), and any cell whose removal materially changes the K-S statistic is treated as an influential point and reported as a separate stratum or excluded with explicit justification, instead of being silently pooled.

Descriptive statistics of the combined 66 valid samples: mean capacity 0.9454 Ah, standard deviation 0.3195 Ah, median 0.8520 Ah. The mean is greater than the median, indicating an obviously right-skewed distribution as a whole, which fully matches the aging law of lithium-ion batteries: slow decay in the early stage and abrupt cliff-like capacity fade in the late stage. Symmetric distributions such as normal and lognormal distributions are difficult to characterize this right-skewed degradation feature, while the two-parameter PRD adopted in this paper can flexibly adapt to right-skewed lifetime data through the shape parameter, so the modeling choice is reasonable.

### 4.2. Goodness-of-Fit Verification of PRD on Complete Samples

Based on all 66 complete samples, the multi-start L-BFGS-B optimization algorithm is used to solve the MLE of PRD, obtaining the scale parameter $\hat{\theta }=0.7700$ and the shape parameter $\hat{\beta }=1.5989$. Since $\hat{\beta }>0.5$, the PRD hazard rate function increases monotonically, which fully matches the microscopic mechanism of lithium-ion battery cyclic cumulative aging and sharply increases late-stage failure risk.

The K-S goodness-of-fit test is performed on the complete samples. The test statistic *D* = 0.1641, corresponding to *p* = 0.0508. This *p*-value is exactly at the critical edge of the 5% significance level. The fit can be deemed acceptable at the 10% significance level but borders on the rejection threshold at the more stringent 5% level.

**Figure 4:**
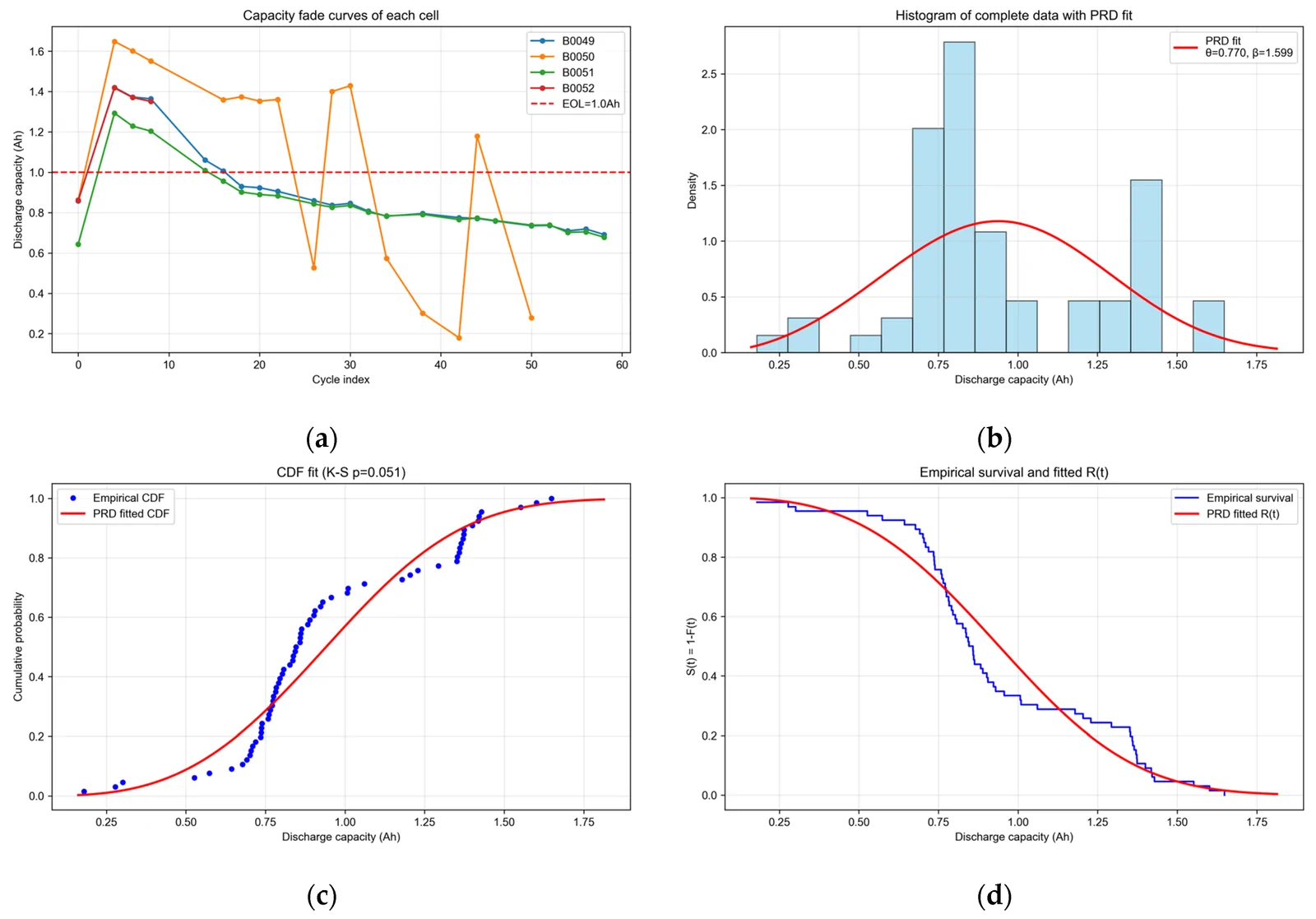

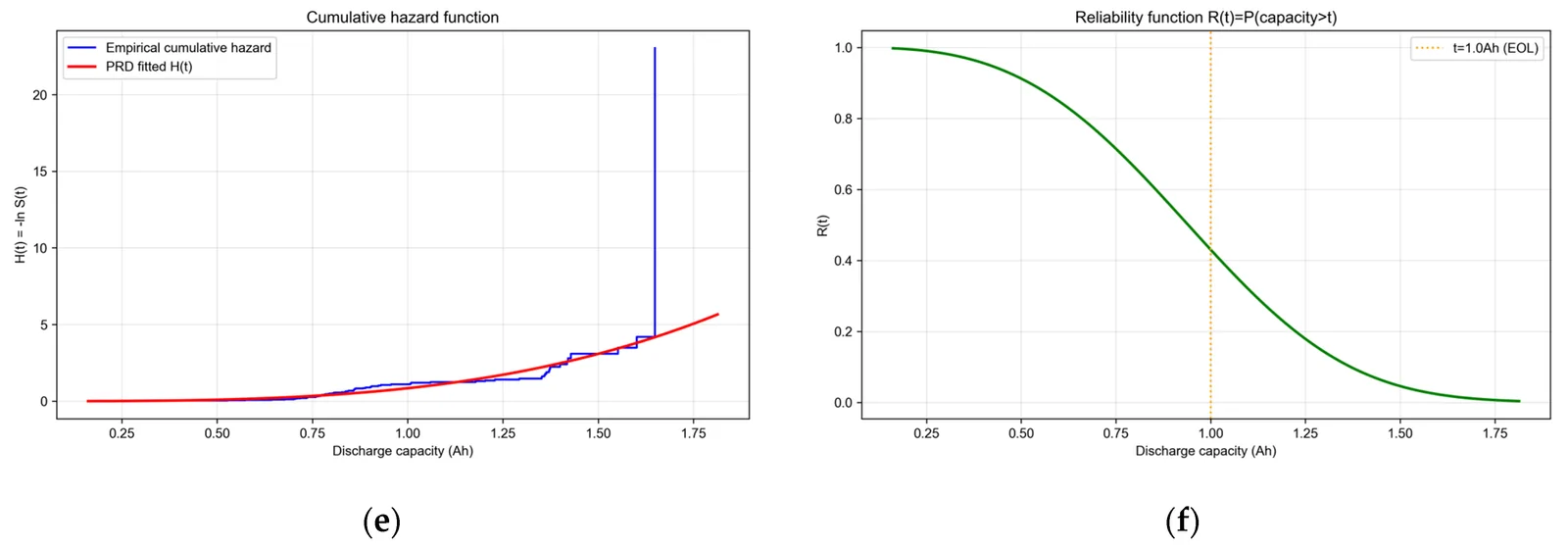
Goodness-of-fit plots for the PRD model on the NASA battery dataset. (**a**) capacity-degradation trajectories with the end-of-life (EOL) threshold marked by a red dashed line; (**b**) sample capacity histogram versus the fitted PRD PDF; (**c**) empirical CDF versus the fitted PRD CDF; (**d**,**e**) empirical reliability and cumulative-hazard functions versus the corresponding PRD theoretical functions; (**f**) reliability as a function of the capacity threshold.

The comprehensive PRD fitting diagnosis is presented in Figure 4, which consists of six panels: (a) marks the end-of-life (EOL) threshold with a red dashed line, where the capacity degradation of the four battery groups exhibits a nonlinear accelerating downward trend; (b) displays the sample capacity histogram, which agrees closely with the PRD theoretical PDF curve in the form of a right-skewed distribution; (c) shows that the empirical cumulative distribution and the PRD theoretical CDF curve exhibit a high overall degree of fit; (d) and (e) illustrate that the empirical reliability and cumulative hazard curves are well synchronized with the corresponding PRD theoretical functions; and (f) demonstrates that the reliability function decreases monotonically as the capacity threshold increases, providing a benchmark for subsequent censoring estimation.

### 4.3. Design of Adaptive Hybrid Censoring Test Schemes

APT-II censoring is implemented post hoc on the complete NASA dataset. Based on 66 sets of complete battery degradation samples, replicating the engineering accelerated aging test scenario, two sets of adaptive censoring comparative schemes with different early removal intensities are designed. The unified basic test parameters are as follows: total sample size *n* = 66, target number of failure observations *m* = 40, capacity shutdown threshold *T* = 0.8 Ah, and total number of reserved samples to be censored *n* — *m* = 26.

The adaptive rule of the CS-I scheme (with mild removal): two surviving units are removed after the 1st and 22nd failures, respectively, and the remaining samples are removed all at once after the last failure. This scheme removes a small amount in the early stage, and is suitable for test scenarios with high estimation accuracy requirements and loose test duration constraints in engineering.

The adaptive rule of the CS-II scheme (with heavy removal): eight surviving units are removed after the 1st and 27th failures, respectively, and the remaining samples are removed all at once after the last failure. This scheme features substantial sample withdrawal in the early test phase, corresponding to the engineering demand for rapidly reducing samples and shortening the test cycle.

Set the adaptive adjustment rule: when the observed failure capacity crosses the threshold *T* = 0.8 Ah, stop the early removal operation, and the remaining units to be removed are accumulated and removed uniformly at the time of the last failure. Based on the actual degradation data, the mid-term removal of CS-I scheme is retained, and the final centralized removal amount after adjustment is 22; for CS-II scheme, the 27th failure capacity is already higher than the threshold, the mid-term removal is canceled and merged into the final removal, and the final centralized removal amount after adjustment is 18.

From the perspective of sample information, heavy early removal in CS-II will screen out more high-capacity and long-cycle samples in advance, which may lose the degradation information of the right tail of the distribution. Therefore, it is estimated that a larger parameter estimation bias will be generated, which will be verified by the MLE and Bayesian results later.

### 4.4. Analysis of Maximum Likelihood Estimation Results

Taking the MLE results of complete data $\hat{\theta }=0.7700,\;\hat{\beta }=1.5989,\;\hat{R}(1.0)=0.4303$ as the benchmark, and selecting the capacity threshold *t* = 1.0 Ah for reliability evaluation, MLE is performed on CS-I and CS-II censored samples, respectively. The results are shown in Table 3.

Both censoring schemes show underestimation of the scale parameter, overestimation of the shape parameter, and significant underestimation of reliability. This is primarily because adaptive censoring only retains early failure samples with capacity below 0.8 Ah, losing the degradation information of high-capacity and long-cycle cells, resulting in the lack of right-tail information of the distribution.

Comparing the two schemes, all parameter estimates of CS-I are closer to the complete sample benchmark, and the reliability estimation bias is significantly smaller than that of CS-II, which proves that mild early removal can effectively reduce information loss. In accelerated aging tests, if high accuracy of reliability evaluation is required, CS-I-type low early-removal censoring schemes should be selected; if test cycle constraints are prioritized, the CS-II scheme can be used, but it needs to be matched with a Bayesian method to correct the estimation bias.

### 4.5. Multi-Loss Bayesian Estimation and Convergence Diagnosis Based on MCMC

Selecting the non-informative prior and M-H MCMC sampling, the iteration settings are 12,000 total steps, burn-in 2000, and 10,000 effective samples. Bayesian estimates are solved under three criteria: squared error loss, generalized entropy loss and LINEX loss. The results are shown in Table 4.

The following conclusions can be drawn from Table 4:

- The estimates from SE and LINEX losses are highly close: for the scale parameter *θ* and reliability *R*(1.0), the relative differences are within 1%; for the shape parameter *β*, the differences are slightly larger (about 1.5–1.8% in the censored schemes), yet the overall agreement remains strong, making both suitable for conventional lifetime assessment scenarios.
- Generalized entropy loss imposes a stronger penalty on parameter overestimation, and the reliability estimation result is the lowest and most conservative, which is suitable for high-safety constraint engineering scenarios such as power batteries and energy storage power stations.
- Bayesian estimation and MLE have consistent bias directions, but the Bayesian method can fully quantify the estimation uncertainty through the posterior distribution, addressing the limitation that MLE only provides a single point estimate.
- Under the same estimation method, the scale parameter and reliability estimates of CS-I scheme are higher than those of CS-II, while the shape parameter is slightly lower than that of CS-II, which again verifies the information retention advantage of mild early censoring.

**Figure 5:**
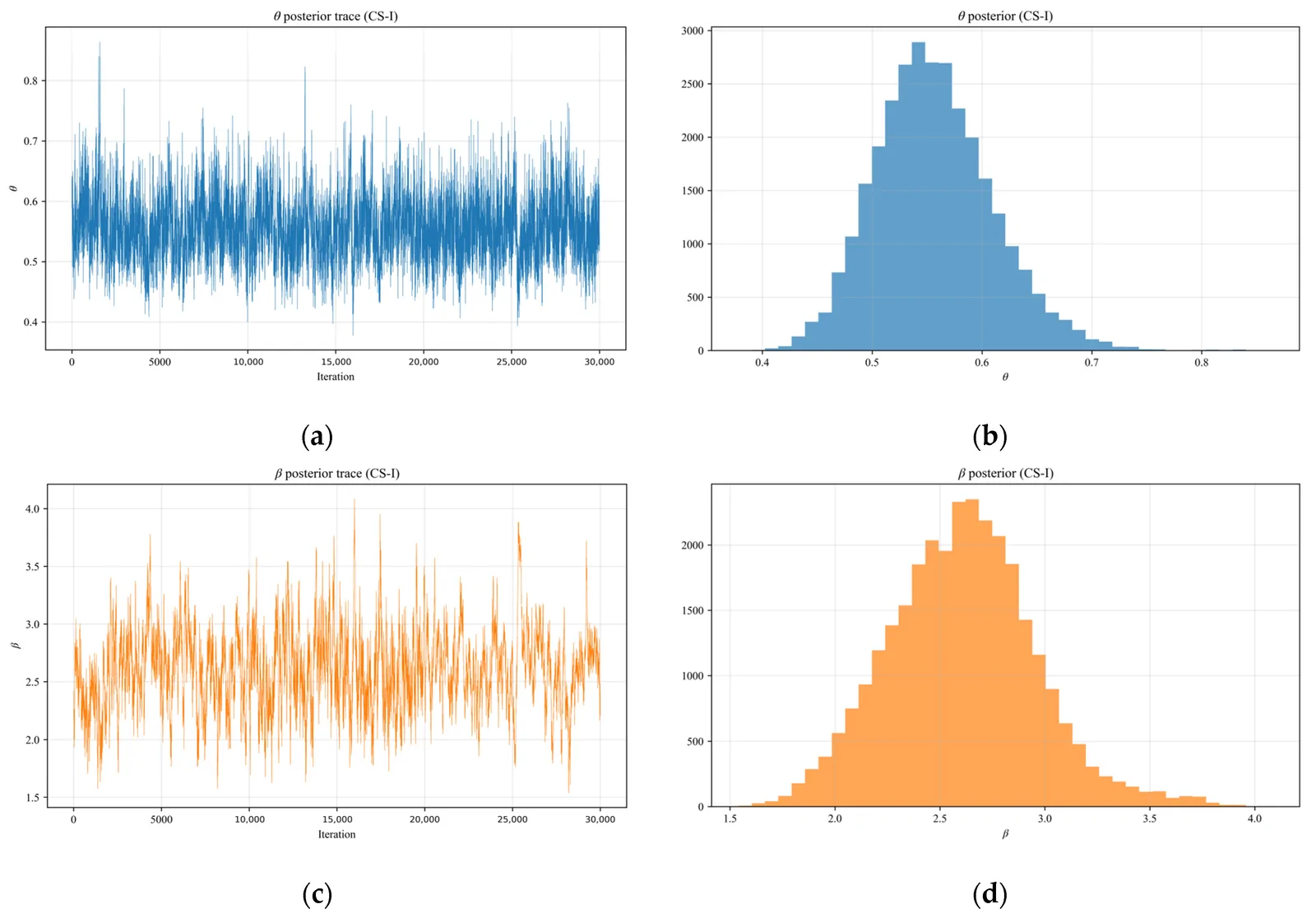
Trace plots and posterior histograms of PRD parameters under Scheme CS-I. (**a**) trace plot of *θ*; (**b**) posterior histogram of *θ*; (**c**) trace plot of *β*; (**d**) posterior histogram of *β*.

**Figure 6:**
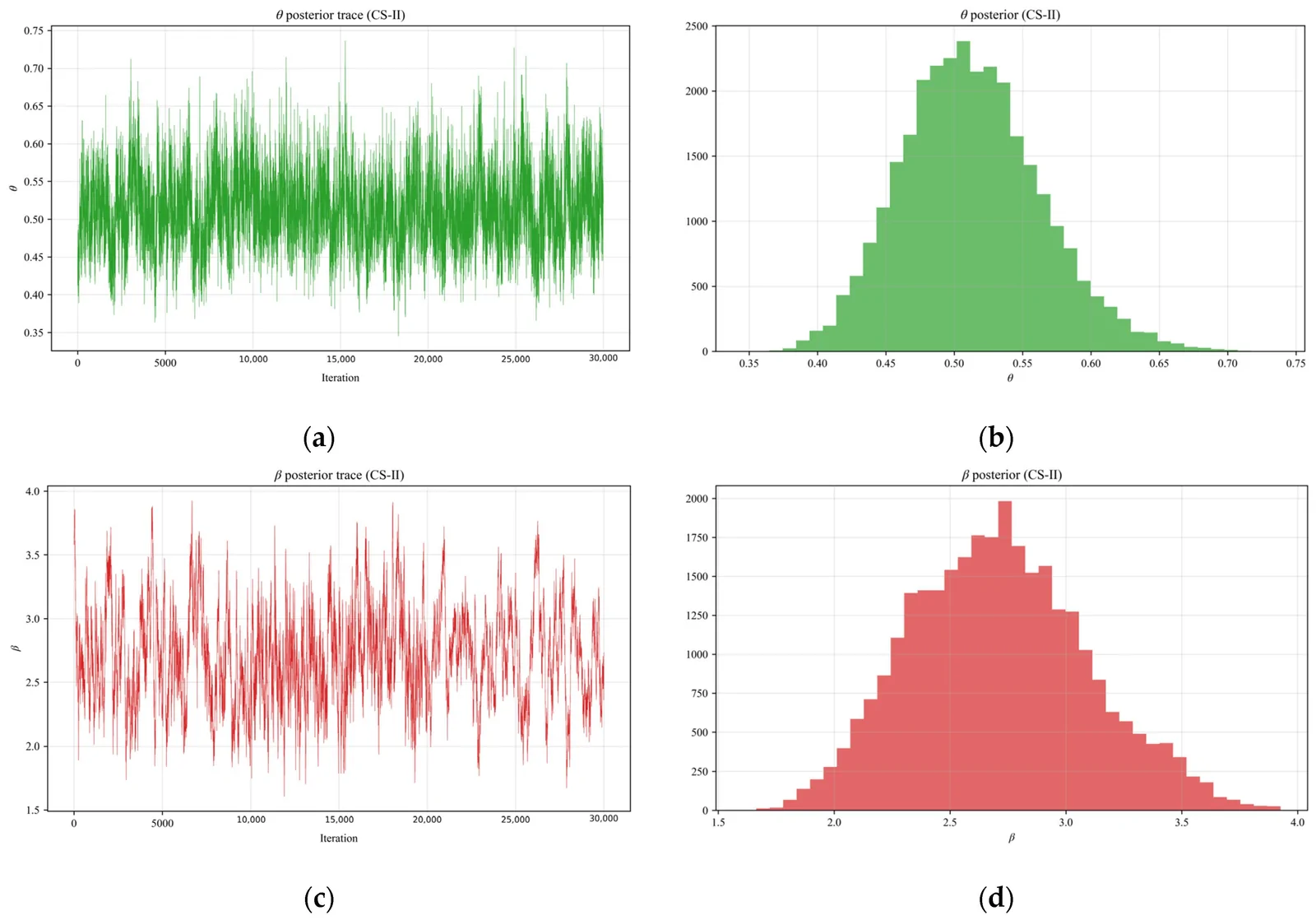
Trace plots and posterior histograms of PRD parameters under Scheme CS-II. (**a**) trace plot of *θ*; (**b**) posterior histogram of *θ*; (**c**) trace plot of *β*; (**d**) posterior histogram of *β*.

The parameter MCMC trace plots and posterior distribution histograms of the CS-I and CS-II schemes are shown in Figure 5 and Figure 6, respectively. The parameter traces in the figures show no persistent drift or periodic trend, and the parameter posterior distributions present a stable unimodal shape, indicating good convergence of the sampling chain. The sampling acceptance rates of both schemes fall within the optimal interval, the standard deviation of the proposal distribution is set reasonably, and the posterior sampling is effective.

### 4.6. Reliability Assessment and Interval Estimation

Taking the reliability *R*(1.0) at the capacity threshold *t* = 1.0 Ah as the evaluation object, ACI, BCI and Bayesian HPD are constructed. The upper and lower bounds and widths of the intervals are shown in Table 5.

By analyzing the interval comparison, it is found that the HPD width is the minimum among the three types of intervals under both schemes, with the best compactness, which can make full use of the complete information of the parameter posterior distribution. BCI has the largest interval width, and the bootstrap resampling mechanism will introduce additional random fluctuations on the basis of censored samples, resulting in the highest estimation uncertainty.

The ACI and HPD widths of CS-II are smaller than those of CS-I, which does not mean higher estimation accuracy. Instead, the heavy early censoring of this scheme concentrates the samples in the low-capacity interval, resulting in smaller variance of reliability values. The reliability curve decreases monotonically with the increase in the capacity threshold and decays faster in the low-capacity interval.

In engineering reliability quantitative analysis, HPD is preferred, and ACI can be used for auxiliary comparison when the sample size is sufficient. BCI is not recommended for small-sample and heavily censored scenarios. It should be pointed out that the interval width ranking under the above measured single-group sample comes from the bootstrap degradation effect under limited samples; in the large-scale Monte Carlo simulation in Section 5.5, due to the average of repeated resampling, the bootstrap interval is instead the narrowest but the coverage probability is significantly lower. Therefore, this measured conclusion does not represent the width ranking under the simulation caliber.

### 4.7. Sensitivity of the PRD Fit to the Influential Cell B0052

B0052 contains only four valid cycle observations, which is the cell with the smallest sample size in the dataset. Its observations are located at the far-right tail of the empirical distribution, which has a potential amplifying effect on the overall fitting shape. To evaluate the sensitivity of this single point to PRD fitting and censoring estimation, the complete procedure is re-executed on the remaining 65 samples after removing B0052. The results are summarized in Table 6.

The sensitivity analysis shows that after removing B0052, the shape parameter *β* under both censoring schemes jumps sharply from about 2.59/2.63 to above 4.20, and the K-S test *p*-value drops sharply from a marginally significant 0.0508 to 0.0000, and the goodness-of-fit is clearly rejected. This indicates that the single cell B0052 has a non-negligible influence on shape parameter estimation and overall fitting conclusion of PRD. The current PRD fitting is already critical at the strict 5% level, and the conclusion is sensitive to extreme small-sample points. Therefore, although the PRD inference framework obtained from the complete 66 samples has been verified to be robust at the simulation level, the measured fitting conclusion still needs to be further confirmed after expanding the sample size. In engineering applications, it is not advisable to make overly deterministic judgments on the battery lifetime distribution shape based solely on the current small-sample fitting results.

### 4.8. Censoring Estimation Bias Analysis

Taking the MLE true value of the complete sample as the benchmark, the relative estimation efficiency RE is used to quantify the bias level of various parameters, estimation methods and censoring schemes. The closer RE is to 1, the smaller the estimation bias. The results are shown in Table 7.

It can be seen from the table that the parameter estimation accuracy is clearly stratified. The scale parameter has the best overall estimation accuracy, while the shape parameter is more sensitive to distribution shape constraints and has larger censoring bias. As a nonlinear composite index of two parameters, the exponential operation of reliability will superimpose and amplify the estimation errors of both types of parameters, resulting in the highest bias level. The estimation performance of different loss functions is clearly distinguished: generalized entropy loss has a stronger penalty for parameter deviation values, corresponding to larger reliability estimation bias and the most conservative output; the estimation accuracy of squared error and LINEX loss is similar, with a difference of less than 2%. Comparing the two censoring schemes, the relative estimation efficiency of CS-I is always better than that of CS-II under the same estimation method, and the relative efficiency of reliability index is about 10 percentage points higher, which proves that the CS-I scheme with mild early removal can achieve the optimal balance between reducing the test scale and ensuring estimation accuracy.

## 5. Model Comparison and Engineering Applicability

### 5.1. Goodness-of-Fit Comparison with Traditional Lifetime Distributions

**Figure 7:**
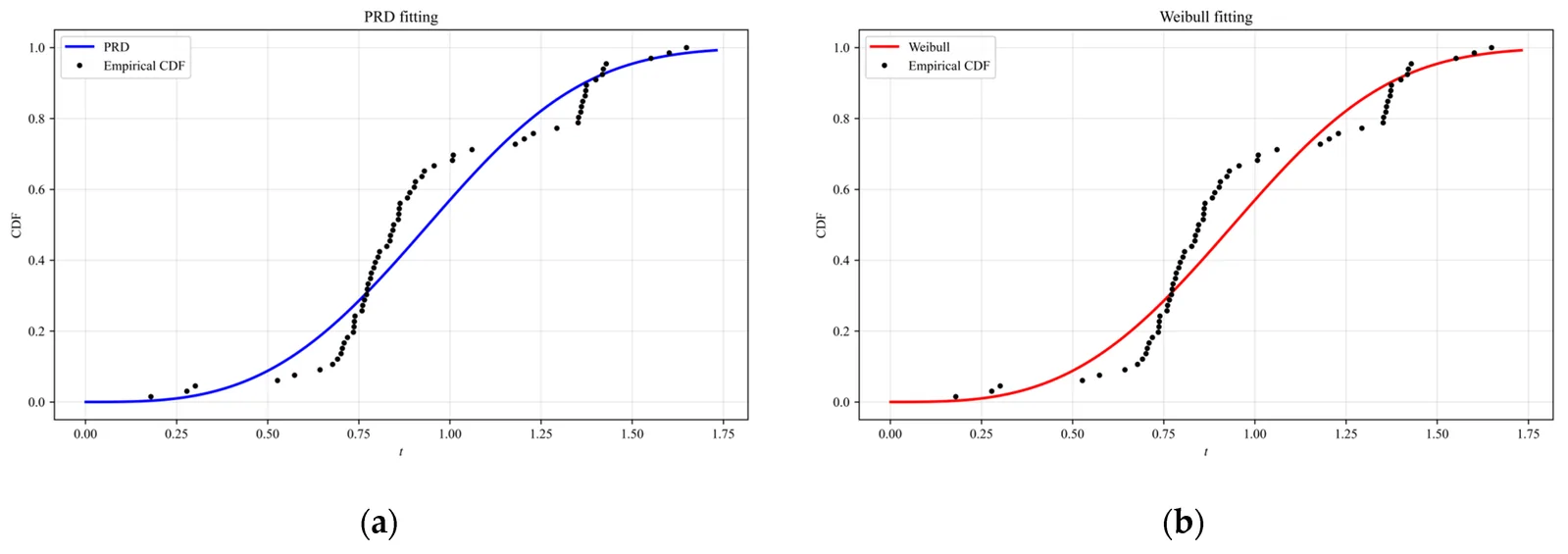

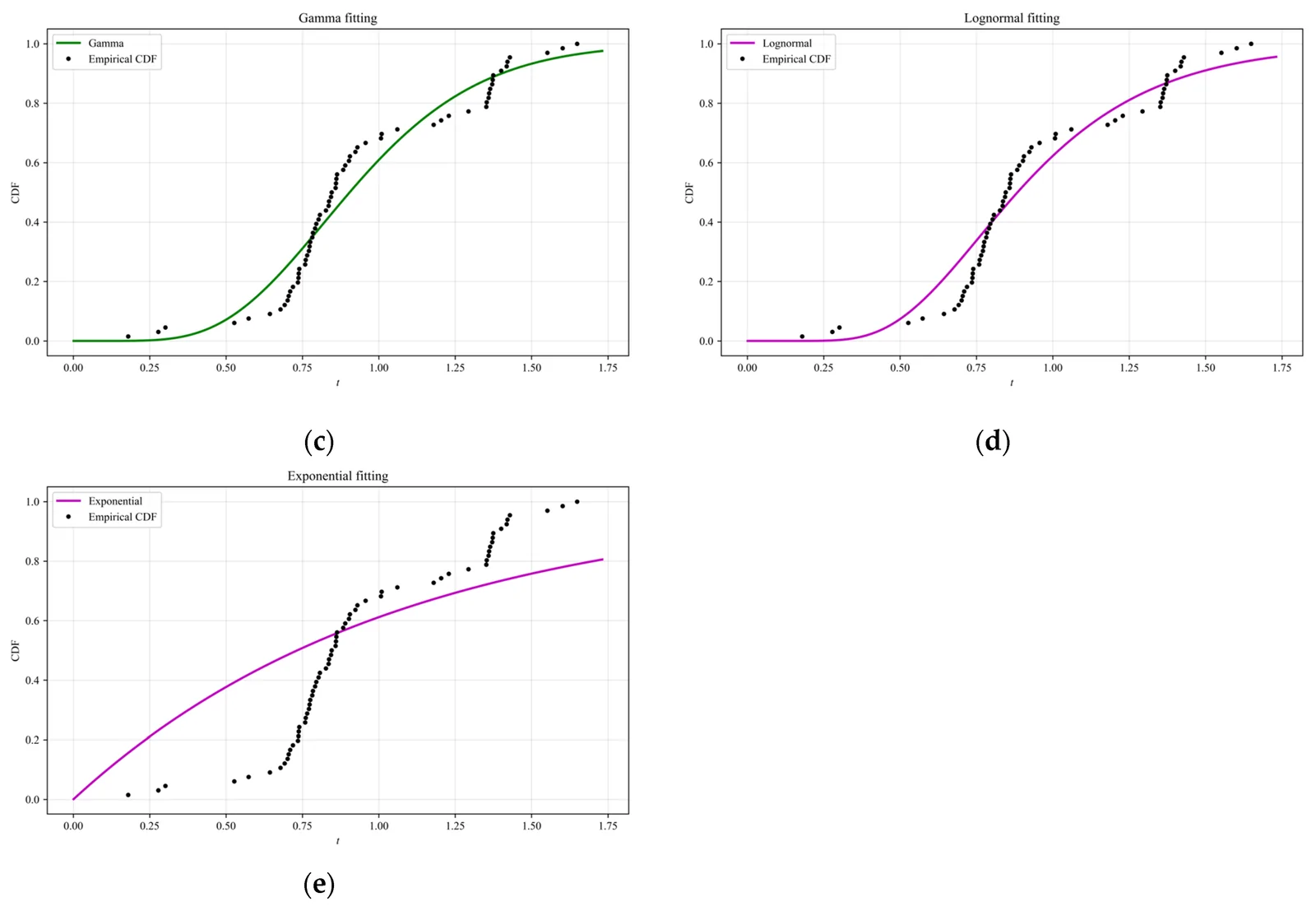
Goodness-of-fit comparison of five lifetime distributions: (**a**) power Rayleigh, (**b**) Weibull, (**c**) Gamma, (**d**) lognormal, (**e**) exponential.

To verify the modeling behavior of PRD for lithium-ion battery capacity degradation data, five lifetime distributions, namely power Rayleigh, exponential, Weibull, Gamma and lognormal, are selected as control models. Fitting is performed on all 66 complete battery samples, and the K-S test, AIC, BIC and log-likelihood values are used as comprehensive evaluation criteria. The comparison results are shown in Table 1 and Figure 7.

The comparison of fitting effects of lifetime distributions is shown in Figure 7.

Combining the table and figures, the following conclusions can be drawn:

- The exponential distribution has a K-S statistic as high as 0.4209 and a *p*-value close to 0, and it is strongly rejected at the 5% significance level; its AIC and BIC are also the worst, indicating that the constant-hazard assumption is inconsistent with the wear-out aging law of lithium-ion batteries.
- The Gamma distribution gives a relatively high K-S *p*-value, but its AIC and BIC are higher than those of PRD and Weibull, so its additional complexity does not translate into a clear generalization advantage, and its parameters are less physically interpretable.
- The lognormal distribution provides a competitive empirical fit, with a K-S statistic of 0.135, *p*-value of 0.17, AIC of 42.6 and BIC of 47.0 (Table 1); it is slightly worse than PRD/Weibull but comparable to Gamma. However, it does not provide elementary closed forms for the hazard rate, Shannon entropy and quantile function, and therefore it does not offer the same computational convenience for repeated Bayesian inference and online PHM computation.
- PRD and Weibull are completely equivalent in terms of AIC, BIC, log-likelihood and K-S test, because PRD is a reparameterization of the two-parameter Weibull family. They coincide when the Weibull shape parameter *k* = 2*β* and the scale parameter $\lambda ={\left(2\theta^{2}\right)}^{\frac{1}{2\beta }}$.
- Because the two models are reparameterizations of one another, no goodness-of-fit statistic can distinguish them (Table 1); the practical difference is analytical tractability and computational cost, not fit. We quantified this by benchmarking the Shannon-entropy evaluation that the generalized-entropy Bayesian loss calls at every posterior draw: the elementary closed form takes about 1.1 µs per evaluation, whereas numerical integration of −*f*(*x*)log *f*(*x*) over [0, ∞) takes about 828 µs per evaluation, a roughly 740× speed-up, and both return the same value (0.4855). Over the 10,000 retained MCMC draws in each CS-I/CS-II chain, this reduces the entropy-loss evaluation from about 8.3 s to about 0.011 s, and the closed form is globally stable for any shape parameter *β* > 0, whereas series/quadrature alternatives carry convergence-interval constraints. PRD is therefore preferred here not because it fits better but because the reparameterization exposes elementary closed forms for entropy and quantiles, lowering the cost and improving the numerical stability of repeated Bayesian and online PHM computation.

Overall, PRD achieves a good balance between fitting accuracy and model simplicity, and it has the advantages of analytical reliability, closed-form hazard rate and direct calculation of Shannon entropy, which is more suitable for Bayesian inference and fast engineering calculation under adaptive censoring scenarios.

### 5.2. Performance Comparison of Censoring Schemes and Parameter Sensitivity Analysis

The core difference between CS-I and CS-II is essentially the different retention degree of the right-tail information of the distribution. The CS-II scheme removes a large number of intact samples in the early stage of the test, and it actively discards the degradation data of long-life and high-capacity cells, resulting in decreased fitting accuracy of the right tail of the distribution. The final performance is underestimation of the scale parameter *θ*, overestimation of the shape parameter *β*, and conservative reliability results. However, this scheme can advance the test termination time by 25–35%, significantly reducing the test duration and sample cost, and it is suitable for rapid verification scenarios in the R&D stage. By contrast, the CS-I scheme only eliminates a small number of samples at the beginning and end of the test. It fully preserves core mid-range degradation data with minimal loss of right-tail information. Its estimation results are closer to the benchmark obtained from complete samples, making it appropriate for accurate reliability assessment at the product validation stage.

The value of the shape parameter *β* has a significant impact on estimation accuracy: when *β* > 1.5, the right-skewness of the lifetime distribution weakens, and the bias caused by censoring will be amplified. At this time, it is more recommended to match the CS-I scheme with Bayesian estimation to correct the bias. When *β* is in the range of 0.5–1.5, the right-skewed feature of the distribution is obvious, and even with the CS-II scheme, the estimation bias is within an acceptable range, so test efficiency can be prioritized.

The selection of reliability threshold also affects the error level: the low-capacity threshold is in the main interval of the distribution, the censored samples are fully covered, and the estimation bias is small; the high-capacity threshold is in the right tail of the distribution, with more missing censoring information and significantly increased bias. In engineering, when evaluating high-reliability indicators, the censoring ratio should be appropriately reduced and the number of effective observation samples should be increased.

### 5.3. Applicable Scenario Analysis of Different Estimation Methods

This paper covers both frequentist and Bayesian point and interval estimation methods, each with distinct pros and cons. Based on prior empirical and simulation results, this section systematically compares them to guide engineering method selection.

For point estimation, MLE offers the highest efficiency and accuracy under moderate samples (*n* = 66) and mild censoring, needs no prior, and suits batch tests with sufficient data. Bayesian estimation under squared-error or LINEX loss achieves comparable accuracy while outputting the posterior to quantify uncertainty, ideal for reliability and life prediction requiring risk intervals. Under generalized entropy loss, Bayesian estimation penalizes overestimation more, giving conservative outputs, suitable for high-safety applications like power batteries to avoid overestimated reliability risks.

For interval estimation, the asymptotic confidence interval is simplest but relies on large-sample normality, performing well with sample size and censoring < 30%, yet coverage deviates under heavy skewness and censoring. BCI is assumption-free but computationally heavy, and resampling cannot preserve APT-II structure, yielding low coverage under heavy censoring; it is only auxiliary. The HPD interval, from the Bayesian posterior, achieves optimal compactness with near-nominal coverage, adapts to asymmetric posteriors, and is preferred for lithium-ion battery reliability, especially for small samples and heavy censoring.

### 5.4. Engineering Application Implementation Suggestions

In the accelerated aging test link, the censoring scheme should be flexibly selected according to the test cycle requirements and accuracy requirements. If the cycle is tight, a heavy removal scheme such as CS-II should be adopted, combined with Bayesian estimation to correct the bias; if accuracy is prioritized, a mild removal scheme such as CS-I should be adopted. At the same time, according to the expected value of the shape parameter *β*, the ratio of the number of effective failures *m* to the total sample size *n* can be optimized to find the optimal balance between test cost and estimation accuracy.

In the battery health assessment link, the parameter estimation method is embedded into the PHM system, taking the cyclic discharge capacity data as input, updating the PRD parameters in real time, and outputting the reliability and remaining useful life results under different capacity thresholds. Relying on the closed-form analytical advantage of PRD, millisecond-level fast calculation can be realized to meet the real-time requirements of online assessment.

In the preventive maintenance decision-making link, differentiated maintenance thresholds should be set based on the reliability assessment results combined with the failure costs of different scenarios. For example, a reliability of 0.7 can be set as the maintenance warning threshold for energy storage power stations and 0.5 for consumer electronics. By precisely optimizing the maintenance timing, the operation and maintenance cost and failure risk can be balanced.

### 5.5. Monte Carlo Simulation Verification

To exclude the random interference of measured data and verify the statistical performance of the method under controlled variable conditions, this section uses Monte Carlo simulation tests to systematically evaluate the bias, root mean square error and interval coverage of various methods under different sample sizes and censoring schemes under the condition of known true values.

Three sets of benchmark true value parameters are set, (*θ*, *β*) = (0.5, 2.0), (1.0, 1.5) and (1.5, 1.0), covering distribution shapes under different shape parameters. Two sets of sample sizes are set: a moderate-sample group (*n* = 66) and small-sample group (*n* = 40). Two censoring schemes CS-I and CS-II are adopted. The comparison methods include three types of point estimation (MLE, squared error/generalized entropy/LINEX loss Bayesian estimation, bootstrap method) and three types of interval estimation (ACI, BCI, HPD). The simulation is repeated 500 times, and bias, RMSE, interval coverage probability and average interval length are used as evaluation indicators.

The point estimation accuracy results under 12 complete configurations formed by the intersection of three groups of true values, two groups of sample sizes and two sets of censoring schemes are shown in Table 8 and Table 9.

The simulation results verify the robustness of the previous empirical conclusions: First, the point estimation accuracy of the CS-I scheme is better than that of the CS-II scheme under all configurations, and mild censoring retains more information. This advantage still holds under the controlled variable environment. Second, in terms of bias, the three Bayesian estimators are not inferior to or even slightly better than MLE in most configurations, but under small-sample and high-skewness configurations, the shape parameter bias of Bayesian estimation is slightly larger than that of MLE. In terms of mean square error, MLE is slightly superior under moderate samples and light censoring, while under small samples and heavy censoring, Bayesian estimation gradually shows its stability advantage by virtue of the complete quantification of uncertainty by the posterior distribution. Third, the bias of the bootstrap method is significantly amplified under heavy censoring and small-sample scenarios. This is primarily because that resampling is difficult to strictly maintain the APT-II censoring structure, so it is not suitable for high censoring ratio scenarios.

The interval estimation performance results under this configuration are shown in Table 10 and Table 11.

The interval estimation results show that on the premise of comparable coverage up to standard, HPD has better compactness than ACI, with shorter average interval length. This advantage exists stably in most configurations. However, in terms of coverage probability, ACI is closer to 95% in most configurations, and HPD is slightly lower under small-sample strong skewness configurations. Comprehensively considering compactness and coverage probability, HPD is still the preferred interval estimation method for lithium-ion battery lifetime evaluation, but attention should be paid to the slight insufficiency of coverage probability under small-sample and strong-skewness configurations in application.

## 6. Conclusions and Prospects

This paper establishes a complete analysis framework that integrates frequentist MLE and Bayesian MCMC inference for reliability inference of lithium-ion batteries under APT-II censoring with the PRD. Based on the electrochemical aging mechanism, it is proved that the PRD hazard rate increases monotonically when the shape parameter *β* > 0.5, which matches the degradation law of battery cyclic cumulative damage and sharply increases late-stage failure risk. Based on the APT-II censoring mechanism, the PRD joint log-likelihood function is derived, two control censoring schemes CS-I with mild removal and CS-II with heavy removal are designed, and the performance of various point and interval estimations is comprehensively evaluated through NASA low-temperature battery measured data and Monte Carlo simulations.

From the frequentist perspective, the multi-start L-BFGS-B algorithm is used to solve the MLE of PRD parameters, and the ACI of parameters and reliability is constructed based on the Delta method and the observed Fisher information matrix. Meanwhile, the bootstrap method is introduced to construct confidence intervals that do not rely on the normality assumption. The results show that MLE has high accuracy under light censoring and moderate samples, but systematic underestimation of scale parameters and reliability occurs under heavy censoring. Moreover, BCI fluctuates greatly because it cannot strictly replicate the adaptive removal structure of APT-II, and it is only suitable for auxiliary reference.

From the Bayesian perspective, non-informative joint prior and hybrid M-H MCMC sampling are adopted, and Bayesian point estimates are obtained under three loss functions: squared error, generalized entropy and LINEX, and the HPD with optimal compactness is constructed. Both simulation and measurement consistently show that under moderate samples and light censoring, the point estimation accuracy of MLE is slightly better than the three Bayesian estimators, but under small samples and heavy censoring, the Bayesian method has more advantages in estimation stability by virtue of the posterior distribution’s ability to fully quantify uncertainty. The HPD interval has the shortest length in most configurations and the coverage probability is close to the nominal level. Comprehensively considering compactness and coverage probability, it is the preferred interval estimation method for lithium-ion battery lifetime evaluation.

In terms of censoring scheme and model adaptation, the CS-I scheme retains more right-tail capacity degradation information, and the estimation efficiency for scale and reliability is about 10% higher than that of CS-II. Although CS-II can shorten test duration by 25–35%, its estimation bias is significantly amplified. The goodness-of-fit comparison shows that the PRD and Weibull have equivalent fitting indexes for measured data, but the PRD has closed forms for Shannon entropy, quantiles and hazard rates, offering numerical advantages in Bayesian iteration and online PHM calculation. The K-S test yields a *p*-value of 0.0508, indicating a borderline fit at the conventional 5% level. Empirical and simulation cross-validation confirm the engineering applicability of the proposed methods. The PRD-APT-II framework can be embedded into the battery management system for millisecond-level online output of reliability and remaining useful life, providing a standardized quantitative tool for energy storage health management.

Future work will extend the framework beyond the present single-stress (4 °C low-temperature) validation to accelerated life testing under combined stresses, explicitly coupling temperature and charge/discharge C-rate, so that the PRD–APT-II model can be calibrated on multi-stress test matrices rather than on one low-temperature cycling regime; we will also incorporate covariates such as depth of discharge. Drawing on the competing risk modeling framework under adaptive censoring proposed by Du et al. [24], we will introduce competing risks to distinguish parallel failure paths such as SEI film growth, lithium dendrites, and electrolyte dry-out. Lightweight Bayesian algorithms such as variational inference or Laplace approximation will be developed to meet the real-time evaluation requirements of on-board BMS, further expanding the application scope of the model in complex working conditions and online monitoring scenarios.

## Figures and Tables

**Table 1:** Goodness-of-fit comparison of five lifetime distributions.

Distribution Type	Parameter 1	Parameter 2	Log-Likelihood ℓ	K-S Statistic	*p*-Value	AIC	BIC
Power Rayleigh	0.7700	1.5989	−18.3798	0.1641	0.0508	40.7596	45.1389
Exponential	1.0577	—	−62.2973	0.4209	0.0000	126.5946	128.7842
Weibull	3.1978	1.0548	−18.3798	0.1641	0.0508	40.7596	45.1389
Gamma	7.6302	0.1239	−19.9082	0.1367	0.1545	43.8164	48.1957
Lognormal	−0.1250	0.4405	19.30	0.135	0.17	42.6	47.0

**Table 2:** Descriptive statistics of discharge capacity for the NASA dataset.

Cell ID	Valid Cycles	Mean	Std. Dev.	Capacity Range
B0049	23	0.8985	0.2097	[0.6914, 1.4209]
B0050	16	1.0613	0.5021	[0.1796, 1.6488]
B0051	23	0.8588	0.1717	[0.6435, 1.2932]
B0052	4	1.2503	0.2263	[0.8607, 1.4183]

**Table 3:** Comparison of MLE results for parameters and reliability under different censoring schemes.

Sample Scheme	$\hat{\theta }$	$\hat{\beta }$	$\hat{R}(1.0)$
Complete uncensored sample	0.7700	1.5989	0.4303
CS-I	0.5504	2.5891	0.1920
CS-II	0.5134	2.6292	0.1500

**Table 4:** Comparison of Bayesian estimation results for parameters and reliability under different loss functions.

Scheme	Estimation Method	$\hat{\theta }$	$\hat{\beta }$	$\hat{R}(1.0)$
Complete data	MLE	0.7700	1.5989	0.4303
CS-I	MLE	0.5504	2.5891	0.1920
CS-I	SE	0.5433	2.5942	0.1852
CS-I	GE	0.5389	2.5403	0.1675
CS-I	LINEX	0.5425	2.5476	0.1842
CS-II	MLE	0.5134	2.6292	0.1500
CS-II	SE	0.5041	2.6563	0.1423
CS-II	GE	0.5003	2.6116	0.1259
CS-II	LINEX	0.5034	2.6176	0.1416

**Table 5:** Comparison of three types of interval estimation results for reliability *R*(1.0).

Scheme	Interval Type	Lower Bound	Upper Bound	Interval Width
CS-I	95% ACI	0.0693	0.3146	0.2453
CS-I	95% BCI	0.1113	0.3839	0.2726
CS-I	95% HPD	0.0683	0.3065	0.2382
CS-II	95% ACI	0.0387	0.2613	0.2226
CS-II	95% BCI	0.0231	0.3000	0.2769
CS-II	95% HPD	0.0498	0.2539	0.2041

**Table 6:** Sensitivity analysis of parameter estimates with and without cell B0052.

Index	Original Data	Data After Removal	Change
Sample size *n*	66	65	−1
CS-I *θ*_MLE_	0.5504	0.2702	−0.2802
CS-I *β*_MLE_	2.5891	4.2591	+1.6699
CS-I K-S D	0.1641	0.3571	+0.1931
CS-I K-S *p*	0.0508	0.0000	−0.0508
CS-II *θ*_MLE_	0.5134	0.2672	−0.2462
CS-II *β*_MLE_	2.6292	4.2031	+1.5739
CS-II K-S D	0.1641	0.3639	+0.1999
CS-II K-S *p*	0.0508	0.0000	−0.0508

**Table 7:** Comparison of relative estimation efficiency of estimators under different censoring schemes.

Parameter to Be Estimated	Censoring Scheme	MLE	SE	GE	LINEX
*θ*	CS-I	0.7148	0.7055	0.6999	0.7045
*θ*	CS-II	0.6667	0.6546	0.6497	0.6538
*β*	CS-I	0.3807	0.3775	0.4112	0.4066
*β*	CS-II	0.3556	0.3387	0.3666	0.3628
*R*(1.0)	CS-I	0.4461	0.4303	0.3892	0.4281
*R*(1.0)	CS-II	0.3486	0.3307	0.2925	0.3290

**Table 8:** Estimation accuracy of the scale parameter *θ* under different configurations.

Sample Size	Censoring Scheme	True Value (*θ*, *β*)	MLE Bias	MLE RMSE	Boot Bias	Boot RMSE	SE Bias	SE RMSE	GE Bias	GE RMSE	LINEX Bias	LINEX RMSE
*n* = 66	CS-I	(0.5, 2.0)	−0.0103	0.0542	−0.0144	0.0558	−0.0084	0.0565	−0.0098	0.0568	−0.0091	0.0565
*n* = 66	CS-I	(1.0, 1.5)	0.0021	0.0859	0.0084	0.0898	0.0168	0.0914	0.0149	0.0908	0.0149	0.0906
*n* = 66	CS-I	(1.5, 1.0)	0.0336	0.1977	0.0654	0.2202	0.0670	0.2234	0.0620	0.2201	0.0587	0.2169
*n* = 66	CS-II	(0.5, 2.0)	0.0097	0.0531	0.0052	0.0525	0.0105	0.0551	0.0092	0.0549	0.0098	0.0549
*n* = 66	CS-II	(1.0, 1.5)	−0.0207	0.0796	−0.0152	0.0817	−0.0054	0.0813	−0.0072	0.0812	−0.0072	0.0810
*n* = 66	CS-II	(1.5, 1.0)	−0.0498	0.1723	−0.0204	0.1782	−0.0125	0.1858	−0.0170	0.1847	−0.0195	0.1830
*n* = 40	CS-I	(0.5, 2.0)	−0.0189	0.0753	−0.0254	0.0778	−0.0166	0.0781	−0.0187	0.0788	−0.0176	0.0783
*n* = 40	CS-I	(1.0, 1.5)	0.0131	0.1262	0.0258	0.1364	0.0400	0.1430	0.0369	0.1414	0.0366	0.1408
*n* = 40	CS-I	(1.5, 1.0)	0.0469	0.2641	0.1106	0.3230	0.1121	0.3097	0.1039	0.3040	0.0981	0.2975
*n* = 40	CS-II	(0.5, 2.0)	0.0084	0.0599	0.0019	0.0597	0.0131	0.0635	0.0110	0.0632	0.0120	0.0632
*n* = 40	CS-II	(1.0, 1.5)	−0.0207	0.1035	−0.0098	0.1091	0.0022	0.1124	−0.0007	0.1116	−0.0008	0.1111
*n* = 40	CS-II	(1.5, 1.0)	−0.0536	0.2355	−0.0018	0.2595	0.0003	0.2487	−0.0066	0.2463	−0.0106	0.2430

**Table 9:** Estimation accuracy of the shape parameter *β* under different configurations.

Sample Size	Censoring Scheme	True Value (*θ*, *β*)	MLE Bias	MLE RMSE	Boot Bias	Boot RMSE	SE Bias	SE RMSE	GE Bias	GE RMSE	LINEX Bias	LINEX RMSE
*n* = 66	CS-I	(0.5, 2.0)	0.0864	0.3280	0.1321	0.3561	0.1265	0.3671	0.1172	0.3629	0.1065	0.3551
*n* = 66	CS-I	(1.0, 1.5)	0.0499	0.2184	0.0832	0.2378	0.0763	0.2339	0.0690	0.2307	0.0647	0.2274
*n* = 66	CS-I	(1.5, 1.0)	0.0422	0.1569	0.0639	0.1685	0.0580	0.1693	0.0532	0.1670	0.0529	0.1661
*n* = 66	CS-II	(0.5, 2.0)	−0.0934	0.2983	−0.0488	0.2952	−0.0505	0.3069	−0.0595	0.3076	−0.0681	0.3058
*n* = 66	CS-II	(1.0, 1.5)	−0.0665	0.2201	−0.0346	0.2200	−0.0382	0.2218	−0.0451	0.2222	−0.0484	0.2209
*n* = 66	CS-II	(1.5, 1.0)	−0.0510	0.1498	−0.0286	0.1474	−0.0324	0.1541	−0.0370	0.1546	−0.0369	0.1538
*n* = 40	CS-I	(0.5, 2.0)	0.1635	0.4700	0.2429	0.5257	0.2367	0.5352	0.2218	0.5267	0.2029	0.5100
*n* = 40	CS-I	(1.0, 1.5)	0.1311	0.3424	0.1891	0.3835	0.1846	0.3892	0.1724	0.3818	0.1637	0.3732
*n* = 40	CS-I	(1.5, 1.0)	0.0500	0.2119	0.0894	0.2352	0.0798	0.2276	0.0723	0.2241	0.0716	0.2222
*n* = 40	CS-II	(0.5, 2.0)	−0.1045	0.3625	−0.0353	0.3658	−0.0591	0.3824	−0.0723	0.3825	−0.0850	0.3783
*n* = 40	CS-II	(1.0, 1.5)	−0.0712	0.2741	−0.0197	0.2714	−0.0298	0.2817	−0.0404	0.2816	−0.0456	0.2787
*n* = 40	CS-II	(1.5, 1.0)	−0.0441	0.1974	−0.0074	0.2021	−0.0186	0.1985	−0.0254	0.1983	−0.0254	0.1968

**Table 10:** Coverage probabilities of interval estimators under different configurations.

Sample Size	Censoring Scheme	True Value (*θ*, *β*)	ACI-*θ*	ACI-*β*	ACI-*R*	BCI-*θ*	BCI-*β*	BCI-*R*	HPD-*θ*	HPD-*β*	HPD-*R*
*n* = 66	CS-I	(0.5, 2.0)	0.936	0.952	0.910	0.700	0.918	0.670	0.902	0.862	0.896
*n* = 66	CS-I	(1.0, 1.5)	0.950	0.962	0.944	0.748	0.944	0.742	0.934	0.924	0.926
*n* = 66	CS-I	(1.5, 1.0)	0.950	0.964	0.924	0.904	0.926	0.874	0.882	0.920	0.872
*n* = 66	CS-II	(0.5, 2.0)	0.958	0.890	0.936	0.664	0.910	0.646	0.926	0.858	0.918
*n* = 66	CS-II	(1.0, 1.5)	0.944	0.924	0.954	0.704	0.906	0.702	0.946	0.910	0.942
*n* = 66	CS-II	(1.5, 1.0)	0.906	0.896	0.954	0.880	0.912	0.880	0.916	0.880	0.928
*n* = 40	CS-I	(0.5, 2.0)	0.914	0.936	0.846	0.596	0.904	0.538	0.880	0.820	0.860
*n* = 40	CS-I	(1.0, 1.5)	0.926	0.958	0.912	0.738	0.928	0.692	0.894	0.872	0.882
*n* = 40	CS-I	(1.5, 1.0)	0.940	0.946	0.922	0.926	0.922	0.870	0.866	0.880	0.856
*n* = 40	CS-II	(0.5, 2.0)	0.974	0.910	0.946	0.664	0.928	0.636	0.938	0.832	0.928
*n* = 40	CS-II	(1.0, 1.5)	0.948	0.912	0.970	0.732	0.914	0.708	0.952	0.894	0.950
*n* = 40	CS-II	(1.5, 1.0)	0.872	0.880	0.930	0.846	0.910	0.838	0.858	0.842	0.858

**Table 11:** Average lengths of interval estimators under different configurations.

Sample Size	Censoring Scheme	True Value (*θ*, *β*)	ACI-*θ*	ACI-*β*	ACI-*R*	BCI-*θ*	BCI-*β*	BCI-*R*	HPD-*θ*	HPD-*β*	HPD-*R*
*n* = 66	CS-I	(0.5, 2.0)	0.2091	1.1719	0.2120	0.1085	1.1728	0.1037	0.1961	1.0314	0.1896
*n* = 66	CS-I	(1.0, 1.5)	0.3327	0.8710	0.1993	0.1936	0.8890	0.1077	0.3237	0.8040	0.1876
*n* = 66	CS-I	(1.5, 1.0)	0.7227	0.5858	0.1602	0.7003	0.5959	0.1361	0.6479	0.5340	0.1380
*n* = 66	CS-II	(0.5, 2.0)	0.2106	1.0740	0.2231	0.1071	1.1127	0.1075	0.1984	0.9725	0.2002
*n* = 66	CS-II	(1.0, 1.5)	0.3206	0.8076	0.2013	0.1769	0.8395	0.1037	0.3127	0.7540	0.1893
*n* = 66	CS-II	(1.5, 1.0)	0.6488	0.5343	0.1661	0.6165	0.5522	0.1407	0.6055	0.4990	0.1465
*n* = 40	CS-I	(0.5, 2.0)	0.2589	1.5313	0.2480	0.1318	1.5942	0.1123	0.2406	1.3110	0.2169
*n* = 40	CS-I	(1.0, 1.5)	0.4379	1.1542	0.2542	0.2900	1.1984	0.1452	0.4248	1.0564	0.2324
*n* = 40	CS-I	(1.5, 1.0)	0.9504	0.7433	0.2048	1.0478	0.7829	0.1733	0.8238	0.6625	0.1681
*n* = 40	CS-II	(0.5, 2.0)	0.2613	1.3450	0.2733	0.1254	1.4126	0.1210	0.2450	1.1582	0.2411
*n* = 40	CS-II	(1.0, 1.5)	0.4124	1.0143	0.2574	0.2617	1.0895	0.1439	0.4029	0.9295	0.2377
*n* = 40	CS-II	(1.5, 1.0)	0.8371	0.6783	0.2138	0.8700	0.7140	0.1785	0.7314	0.6040	0.1762

## Data Availability

The NASA lithium-ion battery dataset used in this study is publicly available; other relevant data are contained within the article.
